# 3D-Printed Vancomycin- and Gentamicin-Loaded Bone Grafts for Osteomyelitis Treatment: Drug Release and Antibacterial Efficacy

**DOI:** 10.1021/acsomega.6c04993

**Published:** 2026-08-03

**Authors:** Oguz Sogut, Umran Aydemir Sezer

**Affiliations:** † 64076 Faculty of Medicine, Department of Pharmacology, Medicine, Medical Device and Dermocosmetic Research and Application Laboratory-IDAL, 32260 Isparta, Turkey; ‡ 52994 Suleyman Demirel University, Institute of Health Sciences, Department of Regenerative Medicine, 32200 Isparta, Turkey; § Suleyman Demirel University, Natural Products Application and Research Center, 32200 Isparta, Turkey

## Abstract

In this study, the potential of 3D printing technology for patient-adaptable osteomyelitis treatment was determined as well as the effects of antibiotic-loaded grafts on physicochemical properties, thermal stability, antimicrobial efficacy, and cell viability were evaluated. Polycaprolactone (PCL) and nanohydroxyapatite (HA) were used as the backbone of 3D grafts using different models, including Grid, Tri-Hexagon, Zigzag, and Gyroid. Later, gentamicin (10% and 15%), vancomycin (10% and 15%), and their combinations (5%–5% and 7.5%–7.5%) were added to the selected model graft. X-ray diffraction, thermal characterization, Fourier transform infrared spectroscopy (FTIR), and Raman spectroscopy analyses were conducted to characterize the obtained filament before 3D printing. The interaction of drugs and HA within the composite filaments was confirmed by FTIR and Raman spectroscopy. Thermal studies showed that the optimal temperature range for 3D printing did not disturb the structural integrity of the grafts. Cell viability and cytotoxicity assays also confirmed sustained cell viability, as well as those mentioned drug-loaded filaments’ tests showed antibacterial effects against osteomyelitis-causing pathogens (*Staphylococcus aureus*, *Escherichia coli*). This study demonstrated the potential of combining 3D printing technology with localized drug delivery systems to enhance osteomyelitis treatment by providing patient-adaptable scaffold architectures that maximize drug efficacy and minimize systemic side effects, representing a promising strategy that warrants further preclinical validation before clinical translation.

## Graphical abstract

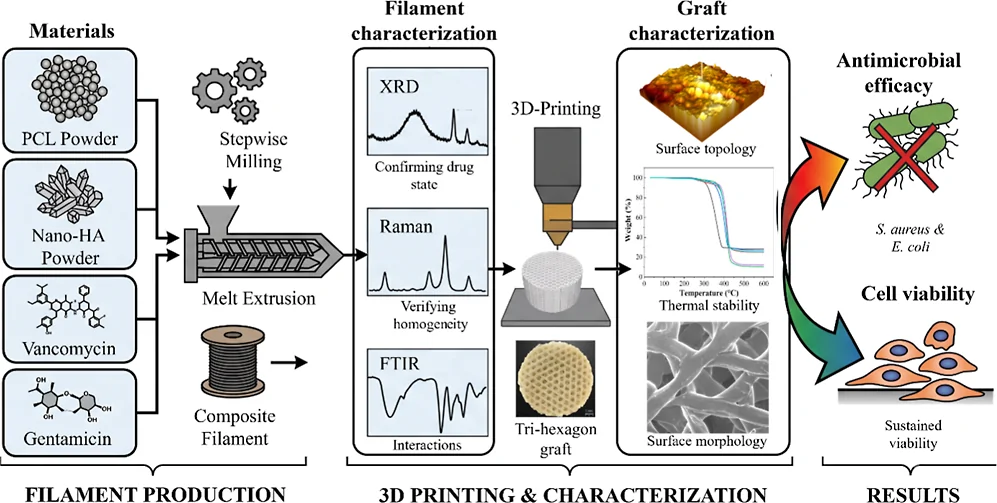

## 1 Introduction

For decades, medical research has struggled greatly with the complicated infection of the bone and bone marrow known as osteomyelitis.^1^ Even with penicillin and other medicines developed in the middle of the 20th century, which greatly lowered related death rates, chronic osteomyelitis remains a challenging and persistent disorder to treat.^2^ With worldwide numbers believed to exceed 2 million, its occurrence in the United States and Europe alone is estimated to be between 300,000 and 500,000.^3^

Osteomyelitis, often triggered by direct bacterial infiltration after trauma or surgery or by hematogenous dissemination, is characterized by increasing bone deterioration and sequestration.^4^ *Staphylococcus aureus* is the most commonly identified pathogen, although chronic cases frequently implicate *Pseudomonas aeruginosa*, *Serratia marcescens*, and *Escherichia coli*.^5^ Bone and joint infections differ from other infectious diseases in their particular physiological and anatomical characteristics, which complicate their clinical presentation and therapy, necessitating tailored treatment approaches.^6^

The main approaches of treatment for osteomyelitis combine pharmacological and surgical actions. While antibiotic therapy addresses systemic infection guided by microbial culture and sensitivity testing, surgical debridement is essential for eliminating necrotic tissue and managing infection.^7^ Long-term, high-dose systemic antibiotic administration, however, sometimes causes serious side effects, including hepatotoxicity and nephrotoxicity. Consequently, localized antibiotic delivery systems have garnered considerable interest as they minimize systemic exposure while also providing higher drug concentrations at the infection site. Antibiotic-loaded spacers, poly­(methyl methacrylate) (PMMA) beads, calcium sulfate pellets, and collagen-based carriers have demonstrated notable improvements in treatment success rates.^8–12^

Underlying systemic diseases including diabetes mellitus, obesity, and vascular insufficiencies further complicate the treatment of osteomyelitis by raising the risk of infection recurrence and treatment failure.^13^ Particularly, chronic osteomyelitis is linked with a 20–30% recurrence probability even with sophisticated therapy approaches. The economic burden of osteomyelitis is significant; the global osteomyelitis market was valued at approximately USD 1.88 billion in 2023 and is projected to reach USD 3.08 billion by 2030, growing at a compound annual growth rate of 7.25%.^14^ The rising prevalence of diabetes and obesity globally means that these expenses will climb as well, which calls for creative and affordable treatment solutions.

Bone tissue engineering (BTE) has emerged as a promising field in response to these challenges by combining modern manufacturing technologies, biomaterials, and cellular therapies to solve bone deformities and infections.^15^ Among these, 3D printing technology has become popular since it can create customized scaffolds that are fit for patient-specific requirements. Comprising polycaprolactone (PCL) and hydroxyapatite (HA), these scaffolds replicate the natural bone matrix and offer a biocompatible platform for localized medication delivery and bone-defect filling.

Owing to its mechanical flexibility, moderate degradation rate, biodegradability, and FDA-approved status, PCL is extensively employed in medical applications.^16^ As a major component of bone minerals, HA improves the mechanical and osteoconductive properties of composite scaffolds, thereby providing an ideal material for BTE.^17^ Incorporating antibiotics such as gentamicin and vancomycin into these scaffolds, researchers aim to produce multifunctional grafts that not only combat bacterial infections but also stimulate new bone formation. Recent research has shown how well 3D-printed bone grafts might achieve regulated medication release.^18–20^ These grafts were engineered to release antibiotics at therapeutic dosages over extended periods, therefore, reducing biofilm development and implant-related diseases. Moreover, the porous architecture of such scaffolds promotes osteogenesis by facilitating nutrient exchange and cellular infiltration.^21^ Furthermore, recent advancements in fused deposition modeling (FDM) have demonstrated that optimizing the physical parameters of PCL/hydroxyapatite composite scaffolds adjusted drug release kinetics while preserving the essential mechanical properties required for load-bearing applications.^22,23^ Nevertheless, optimizing material properties, drug-loading methods, and release kinetics to satisfy clinical needs remains a challenge despite recent advances.^21^ To address these specific challenges, novel strategies such as tailored assembly systems, functionally assembled Lego-like modules, and nanospike-based adhesive materials have recently been designed for the controlled codelivery of multiple therapeutics and structural tunability.^24–26^

The primary innovation of the present study lies in the simultaneous dual-antibiotic loading of a 3D-printed biodegradable PCL/HA composite graft with both vancomycin and gentamicin, enabling broad-spectrum antibacterial coverage against the predominant Gram-positive (*S. aureus*) and clinically relevant Gram-negative (*E. coli*) osteomyelitis pathogens within a single patient-adaptable construct. Unlike prior studies that evaluated single-antibiotic scaffolds or characterized scaffold architecture and drug release in isolation, the present work integrated systematic infill pattern optimization, a validated UHPLC-MS/MS quantification method, release kinetics analysis, antimicrobial efficacy testing, and an ISO 10993-5 biocompatibility assessment within a unified experimental framework. Thus, this work aimed to generate scaffolds with tailored drug release patterns and improved mechanical stability by employing advanced manufacturing processes. The results of this study are expected to contribute to the growing body of knowledge in bioengineering and patient-tailored local drug delivery by providing a novel approach to treating chronic osteomyelitis.

## 2 Materials and Methods

### 2.1 Materials

Polycaprolactone (PCL, *M*_n_ of 80,000 Da) and hydroxyapatite (HA) were supplied by Sigma-Aldrich (USA). Vancomycin (V) and gentamicin (G) were purchased from Sigma-Aldrich (USA) and were incorporated into the composite matrix to achieve targeted antimicrobial activity. Both vancomycin and gentamicin were selected based on their proven efficacy against osteomyelitis-associated pathogens where vancomycin targeted Gram-positive *S. aureus*, while gentamicin targeted Gram-negative *E. coli* and other relevant pathogens. Although *S. aureus* remains the predominant causative agent of osteomyelitis, Gram-negative organisms, including *E. coli*, account for approximately 10–15% of cases, particularly in post-traumatic and hematogenous infections in immunocompromised patients.^27^ Inclusion of a Gram-negative representative, thus provides a broader antibacterial evaluation relevant to mixed-flora scenarios encountered clinically.

Other chemicals used in the experiments, including solvents and analytical reagents, were of analytical or chromatographic grade and were supplied by Sigma-Aldrich (USA).

### 2.2 Preparation of Composite Powder Materials for Filament Production

A stepwise milling was used for PCL under nitrogen gas to avoid agglomeration due to its low glass-transition temperature. Initially (premilling), the particle size of PCL was reduced to less than 500 μm using a homogenizer. Subsequently, a high-speed homogenizer was used to reduce the average particle size to approximately 70 μm. Similarly, HA was ground to resemble the natural range of HA crystals in bone, being 15 to 250 nm, as well as vancomycin and gentamicin, which were milled to obtain ideal particle sizes for integration. Therefore, a premilling step was applied to HA and antibiotics using a high-energy ball mill (Emax, RETSCH, Germany) by dry milling. Subsequently, wet milling using isopropanol as a dispersion medium was used to reduce the average particle size to approximately 200 nm. This procedure included premilling with bigger ZrO_2_ beads (10 mm diameter, 3.5 g), followed by fine milling with smaller beads (0.1 mm diameter, 110 g) at 2000 rpm for different times (30, 60, 120 min).

The characterization analysis (Supporting Information (Characterization of Powders)) and the related results for the produced powders are explained in the Supporting Information (Tables S1 and S2 and Figures S1–S4) in detail.

### 2.3 Preparation of Drug-Loaded Filaments

The drug-loaded composite filaments were prepared by combining PCL, HA, vancomycin, and gentamicin at determined ratios, as shown in Table 1. Varying amounts of vancomycin and gentamicin were used in the drug-loaded filament formulations to compare their antibacterial effects.

**Table 1:** Composition of Composite Filaments^a^ ^,^ ^b^

code	PCL (%, w/w)	HA (%, w/w)	vancomycin (%, w/w)	gentamicin (%, w/w)
control	80	20	-	-
V10	70	20	10.0	-
V15	65	20	15.0	-
G10	70	20	-	10.0
G15	65	20	-	15.0
VG5	70	20	5.0	5.0
VG7.5	65	20	7.5	7.5

a PCL, polycaprolactone and HA, hydroxyapatite.

b All compositions are expressed as nominal weight fractions (w/w) of the total filament formulations.

Powder samples were thoroughly mixed with a Pulverisette 23 homogenizer (Fritsch GmbH, Germany) to achieve a homogeneous blend. The initial combinations were processed to produce filaments with a standard diameter of 1.75 mm using a custom-built single-screw extruder (Türkiye) having a diameter of 25 mm, a length of 650 mm (*L*/*D* ratio of 26:1), and a pitch of 20 mm with a straight-thread configuration. Temperature was selected to be 170 °C at the entrance point and 180 °C at the exit to achieve an appropriate melt viscosity for PCL filament development while retaining the thermal stability of integrated antibiotics. To keep a constant extrusion flow rate, the screw rotation speed was kept at 7 rpm. The filaments were straight and passed into a chilling bath (length of 30 cm and a depth of 2 cm) set at 4 °C cold ethanol after extrusion. Ethanol was chosen as the cooling media since antibiotics have extremely poor solubility in ethanol, which prevented their disintegration and loss during the chilling process. This fast-chilling phase was used to fix the filament structure and to prevent deformation. The cooled filaments were wound with a filament winding machine (Felfil, Spooler+, Italy) to get the required thickness and stability.

The characterization analyses and the related results for the drug-loaded filaments are shown in Supporting Information (Morphological and Chemical Properties; Tables S3–S5 and Figures S5–S12). Characterization analysis was performed to find out any potential chemical changes within the filament materials due to temperature and pressure applied during production and printing. For instance, to simulate actual 3D printing conditions, the drug-loaded filaments were tested for thermal and oxidative stability at 200 °C with TGA (Figures S11 and S12), where XRD (Figure S9 and Table S3), Raman, and FTIR analyses (Figures S7 and S8) confirmed that their chemical and structural integrity remained intact following extrusion. Furthermore, while AFM analysis identified changes in surface roughness due to drug incorporation (Figure S6), the overall results proved that the composite matrix maintained the necessary homogeneity and stability for practical use. Drug-loading efficiency was not directly determined by solvent extraction in the present study. Instead, the theoretical drug content, calculated as the product of the measured graft mass and the nominal drug-loading fraction (w/w) of each formulation, was used as the basis for cumulative release calculations. The composite filament fabrication process employed as a continuous solid-state melt extrusion operation in which all components (PCL, nano-HA, and antibiotic powders) were gravimetrically blended and coextruded without any solvent, precipitation, centrifugation, or filtration step at which drug loss could occur. Mass conservation principles, therefore, dictated that the nominal input composition represented the actual drug content of the resultant filament.

### 2.4 3D Printing of Grafts

Drug-loaded grafts were produced using an extrusion-based 3D printing technique (Dr. INVIVO 4D2 bioprinter, ROKIT Healthcare, South Korea), which was applied with tailored parameters to enhance layer adhesion and structural integrity. To establish the upper limits of printing for the produced drug-loaded composite filaments, the thermal degradation temperatures of PCL, vancomycin, and gentamicin were determined, as explained in thermal characterization. Subsequently, different printing parameters were tested to optimize the 3D printing process, including the printing speed, print head temperature, and build platform temperature, as shown in Table S6. The optimal parameters were determined as 170 and 45 °C with the print rate of 15 mm/s for the print and the print platform, respectively. The pure PCL graft, the PCL/HA graft, and the drug-loaded composite grafts were prepared according to the determined optimal printing parameters. The NewCreatorK V1.57.71 program was used to design implementation, with each layer rotated by 30° to improve interconnection and create irregular voids where cells could adhere (Figure 1). The design included four different infill patterns (Grid, Tri-Hexagon, Zigzag, and Gyroid) with a diameter of 10 mm, a height of 5 mm, and a line width of 400 μm.

**Figure 1:**
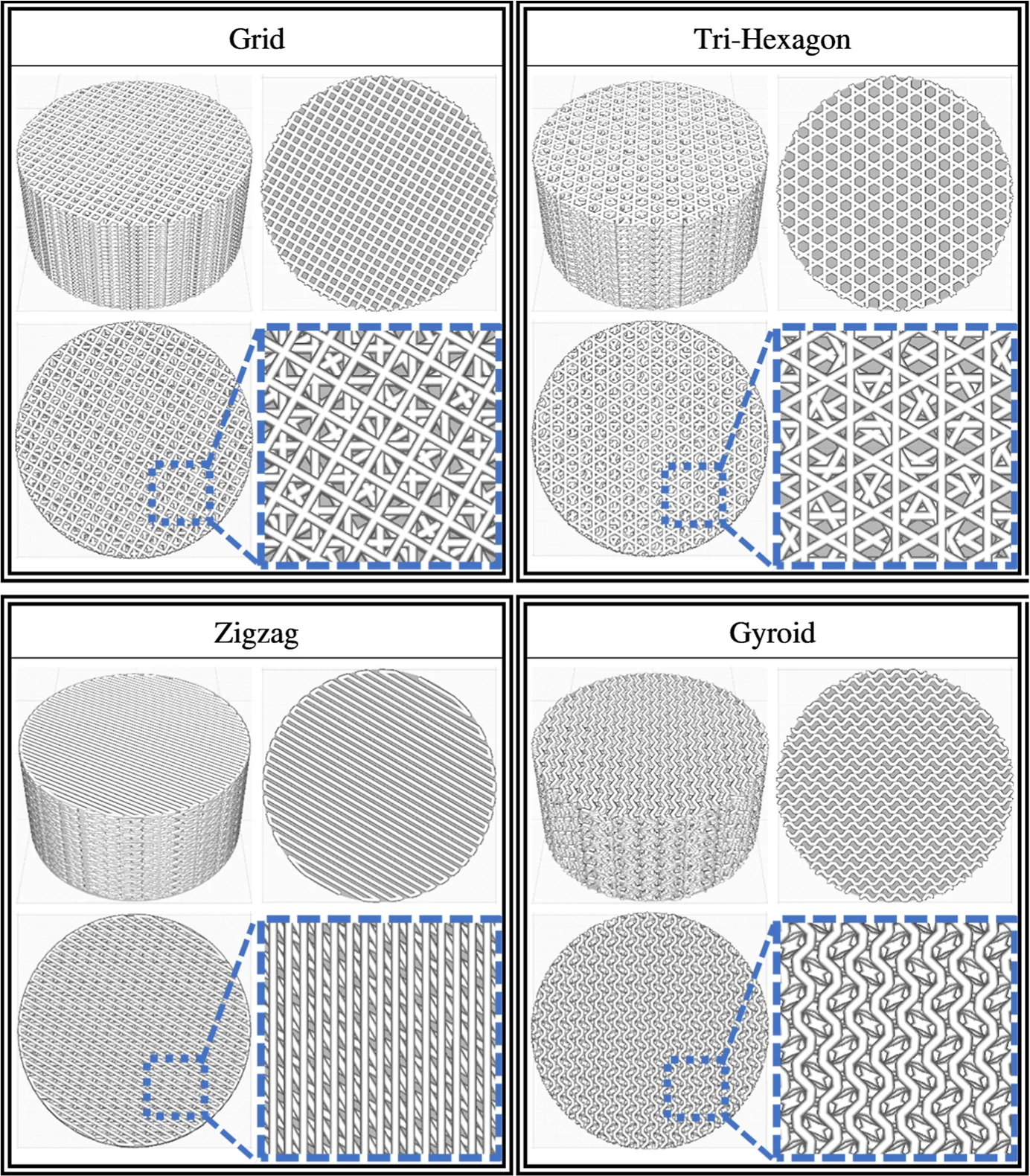
Cylindrical models with four 3D printing infill patterns (Grid, Tri-Hexagon, Zigzag, and Gyroid), presented in top, side, and magnified perspectives to emphasize their unique interior architectures (the STL file of the base cylindrical geometry (10 mm diameter × 5 mm height) used for all graft designs is provided as Supporting Information).

The calculated porosity values for designed grafts showed variations across different infill patterns. The Tri-Hexagon model demonstrated the highest porosity (∼58%), followed by Gyroid (∼57%), Grid (∼56%), and Zigzag (∼55%). These values indicated the impact of internal architecture on void distribution, which could directly influence cell attachment and nutrient diffusion.

### 2.5 Selection of Optimal Pattern for 3D Printing

#### 2.5.1 Morphological Characteristics

The morphological characterization of grafts 3D-printed using different patterns was conducted using a scanning electron microscope (SEM, FEI, QUANTA FEG 250, USA) under low vacuum. The layer-by-layer deposition and pore connectivity of the printed grafts were observed at magnifications of 75× and 100×.

#### 2.5.2 In Vitro Cell Proliferation

In vitro cell proliferation assays were also performed to select the optimal microstructure design for further analysis, including the antimicrobial activity and efficacy in an in vitro cell culture environment and release medium. Briefly, drug-free models were printed and sterilized using ethylene oxide before cell seeding to ensure biocompatibility. Then, human osteoblast (hOB)-like cells (hOBs) were cultured on those 3D-printed grafts with different microstructures (Grid, Tri-Hexagon, Zigzag, and Gyroid). Cell proliferation was evaluated using a resazurin-based viability assay and measuring metabolic activity as an indicator of cell growth. Measurements were taken on the 24th, 48th, and 72nd hours of postseeding.

### 2.6 Characterization of 3D-Printed Drug-Loaded Grafts

#### 2.6.1 Morphological Characteristics

The surface morphology of drug-loaded grafts printed using the selected pattern was observed using SEM, as explained in the Morphological Characteristics section of Methods above.

#### 2.6.2 Antimicrobial Activity of Drug-Loaded Grafts

The antimicrobial efficacy of the selected pattern-based 3D-printed drug-loaded grafts was evaluated using both zone inhibition tests and liquid culture assays against *S. aureus* (ATCC 25923) and *E. coli* (ATCC 25922), with all assays performed in triplicate (*n* = 3 independent biological replicates). The selected cultures were recovered from frozen stocks stored at −80 °C in Brain Heart Infusion (BHI) liquid medium supplemented with glycerol (30%). The strains were subcultured on BHI agar and liquid media at 37 °C for 24 h to ensure active growth. Colonies were suspended in BHI broth and adjusted to a concentration of 8 log CFU/mL using a densitometer (BioSan, DEN-1 McFarland, Latvia).

For zone inhibition tests, the adjusted inoculum was spread evenly on nutrient agar plates. 3D-printed graft samples were then placed onto the inoculated plates and incubated at 37 °C for 20 h. The diameters of inhibition zones were measured using a digital caliper to evaluate the antimicrobial activity of the samples.

For the liquid culture assay, the inoculum was diluted to 8 log CFU/mL and transferred into sterilized tubes containing 10 mL of sterile BHI liquid medium. Preweighed 3D-printed graft samples (4 cm^2^) were added to the tubes and incubated at 37 °C for 24 h. After incubation, serial dilutions were prepared using peptone water, and microbial counts were determined using the pour plate method on BHI agar. The inhibition values of the 3D-printed grafts were calculated by comparing microbial counts in test tubes containing grafts with those in control tubes without grafts. The results were expressed as log CFU/mL, quantifying the reduction in microbial growth caused by the drug-loaded grafts.

#### 2.6.3 In Vitro Release Behavior of Drug-Loaded Grafts and Release Kinetics

The release profiles of the selected pattern-based composite grafts were evaluated at 37 °C under controlled conditions using orbital shaking and temperature control (Heidolph Unimax 1010 combined with Heidolph Incubator 1000, Germany). Phosphate-buffered saline (PBS) at pH 7.4 was used as the release medium to simulate the physiological environment. Release medium was collected at different time intervals and was replaced with fresh PBS. The collected samples were subsequently subjected to quantitative analysis using Thermo Scientific Ultimate 3000 UPLC and Thermo Scientific TSQ Fortis systems utilizing Xcalibur software, both developed by Thermo Fisher Scientific Inc. (Waltham, Massachusetts, USA). For chromatographic separation, a Thermo Fisher Scientific Inc. (Waltham, Massachusetts, USA) Hypersil Gold RP C18 (1.9 μm) UHPLC column, with dimensions of 50 × 2.1 mm, was used. Mobile phase A included 95% water and 5% methanol (containing 0.1% formic acid) and 4 mM ammonium formate. Mobile phase B consisted of 95% methanol and 5% water (containing 0.1% formic acid) and 4 mM ammonium formate. Analytical validation of the UHPLC-MS/MS method used for drug quantification was performed in accordance with ICH Q2­(R1) guidelines. LC-MS/MS method validation was performed in accordance with ICH Q2­(R1) guidelines. Calibration curves were constructed using second-degree polynomial regression over the range of 10–100 μg/mL for both vancomycin and gentamicin; all curves yielded *R* ^2^ > 0.999 (Figure S13). Multiple reaction monitoring (MRM) transitions used for quantification are listed in Table S7. Intraday precision (*n* = 6) was 2.6% RSD for gentamicin and 3.5% RSD for vancomycin; interday precision (*n* = 18) was 5.3% and 7.8%, respectively. Accuracy recovery (*n* = 6) was 97.8% for gentamicin and 101.1% for vancomycin. All values are within ICH Q2­(R1) acceptance criteria. The methods applied for characteristics of release behavior and release mechanism with its kinetics are explained in Supporting Information (In Vitro Release Behavior and Release Kinetics) in detail.

#### 2.6.4 Efficacy of Drug-Loaded Grafts in an In Vitro Cell Culture Environment

The human osteoblast cell line (hOB) was used as the cell model to test the efficacy of selected pattern-based 3D-printed drug-loaded grafts. All grafts were first cultured within a Class II biosafety cabinet (ESCO, Labculture Class II Type A2 BSC, Singapore). Subsequently, samples from these cultures were analyzed using a multimode plate reader (PerkinElmer, VICTOR Nivo, USA). The growth medium consisted of Dulbecco’s Modified Eagle Medium (DMEM) (Thermo Fisher Scientific, Catalog No: 31053044) supplemented with 10% fetal bovine serum (Sigma-Aldrich, Catalog No: F7524), 1% penicillin-streptomycin antibiotic solution (Thermo Fisher Scientific, Catalog No: 15140122), sodium pyruvate (Sigma-Aldrich, Catalog No: S8636), and glutamine (Thermo Fisher Scientific, Catalog No: 25030081). Furthermore, Alamar Blue reagent (Sigma-Aldrich, Catalog No: TOX8-1KT) was employed as a viability indicator. Cell cultures were maintained in a controlled environment to assess the impact of the drug-loaded models on cell behavior, offering insights into the potential therapeutic efficacy and biocompatibility of the composite materials. Therefore, the pH of the culture medium was monitored during the in vitro biocompatibility assays to ensure that grafts and the released drugs did not adversely change the extracellular environment. As high-dose release of gentamicin or vancomycin could potentially induce localized pH shifts, measurements were taken at 24 and 72 h of incubation.

#### 2.6.5 Mechanical Characterization

The compressive mechanical properties of 3D-printed drug-loaded grafts were evaluated using a testing machine (Shimadzu Autograph AGS-X, Japan) equipped with a 10 kN load cell. Cylindrical specimens (*n* = 5 per group) were tested under uniaxial compression at a crosshead speed of 1 mm/min at room temperature in the dry state. Compression was applied up to 55% of the model height, ensuring consistent deformation across all samples. Compressive strength was defined as stress at the 0.2% offset yield point, and a tolerance of ±5% was maintained during the tests where forces exceeding 5 N were recorded. Compressive modulus was then calculated as the slope of the linear elastic region of the stress–strain curve.

### 2.7 Statistical Analysis

The data obtained were analyzed by using GraphPad Prism 6 software. The normality distribution of the data was assessed by using the Shapiro–Wilk test. Parametric data were compared using two-way ANOVA, and nonparametric data were compared using Kruskal–Wallis tests with a significance level at *p* < 0.05. For cytotoxicity data, a prespecified sensitivity analysis excluding the first week of culture (burst-release cytotoxic phase) was planned prior to data collection to evaluate long-term cell viability independent of the initial drug concentration spike.

## 3 Results

### 3.1 Selection of Optimal Pattern Design

Graft models were printed by rotating each layer at 30° angles relative to the previous layer to mimic irregular voids within the body, enhance cell adhesion, and promote viability. Thus, the optimal microstructures for further application were selected using SEM and in vitro cell proliferation analyses. The SEM images of models produced with different fill patterns are shown in Figure 2.

**Figure 2:**
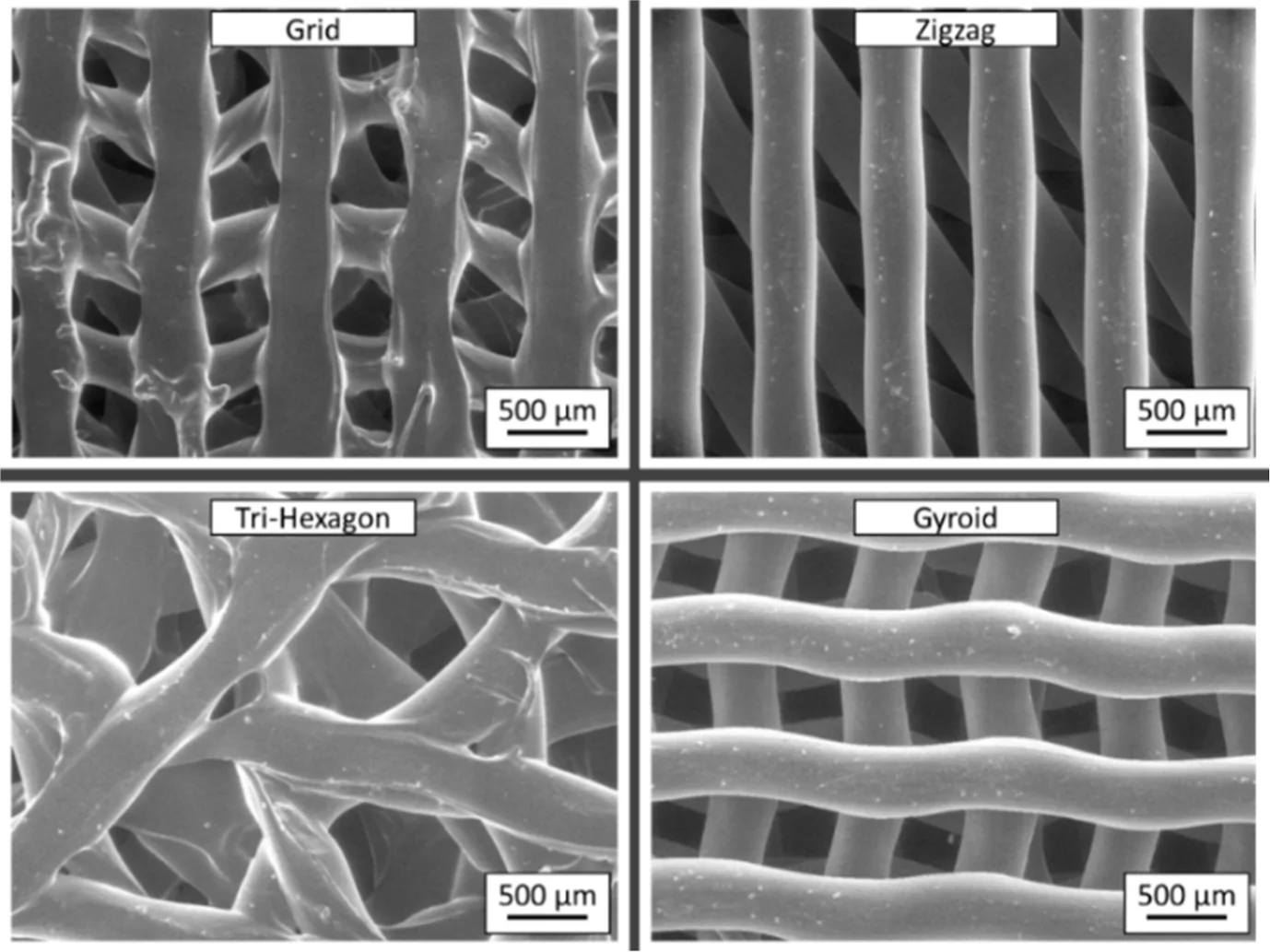
SEM images of models produced with different fill patterns.

The morphological characterization of the drug-free PCL/HA grafts via SEM confirmed the high-precision fabrication of the fill patterns. The SEM images confirmed consistent strand deposition and fully interconnected porous networks, indicating that the optimized printing parameters successfully maintained the desired interconnected structures across various geometries. While the Grid pattern had regular orthogonal pores, the Tri-Hexagon architecture presented a biomimetic, continuous curved geometry, which could be accepted as theoretically superior for promoting cellular colonization due to its high surface area-to-volume ratio. Images also showed a homogeneous integration of HA nanoparticles within the PCL matrix, resulting in a characteristic surface roughness. This rough topography is of significant biological importance, as such microrough surfaces are known to enhance osteoblast adhesion and proliferation compared to smooth polymers.^28^

The effect of fill patterns on the cell viability is shown in Figure 3.

**Figure 3:**
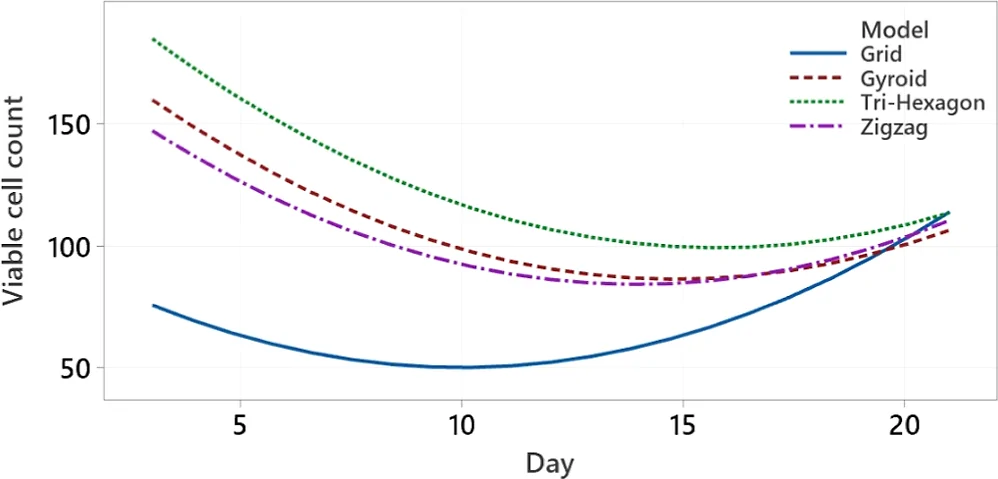
Cell viability based on the fill pattern.

Statistical analysis showed that the fill pattern significantly affected cell viability (*p* < 0.01). Gyroid and Tri-Hexagon patterns supported the highest levels of cell proliferation, attributed to their larger surface area and interconnected porosity. Throughout the 21 day cell culture study, the Tri-Hexagon fill pattern consistently supported the highest number of live cells. Grid and Zigzag patterns had lower cell proliferation rates, likely due to reduced void space and limited surface area. These findings are further corroborated by our previous study on citrate-enhanced PCL scaffolds for bone tissue engineering, where the same four infill patterns (Tri-Hexagon, Grid, Zigzag, and Gyroid) were evaluated with 30°layer rotation.^29^ In that study, the Tri-Hexagon model presented the highest porosity (∼58.36%) among all scaffold designs, which was associated with high cell proliferation rates across all experimental groups, providing additional support for selecting this infill pattern in the present study. Although the absolute porosity values among the four architectures covered a close range (Zigzag ∼55% to Tri-Hexagon ∼58%), it has been well established in the bone tissue engineering literature that even incremental differences in pore geometry and interconnectivity exerted disproportionate effects on cell colonization, nutrient transport, and vascularization. Studies showed that outcomes were more strongly governed by pore architecture than by total porosity alone.^30,31^ The statistical significance of the observed cell viability differences (*p* < 0.05) across the 21 day culture period provided quantitative evidence that the Tri-Hexagon architecture offered a biologically better microenvironment, independent of the modest magnitude of porosity differences. Regarding pore interconnectivity, SEM characterization (Figure 2) confirmed fully interconnected pore networks across all infill patterns, with the Tri-Hexagon geometry exhibiting a continuous, splitting strand arrangement suitable for nutrient diffusion pathways. Following the SEM characterization, cell proliferation assays demonstrated that the Tri-Hexagon geometry offered the most conducive microenvironment for osteoblast attachment, spreading, and proliferation compared to the other designs. The convergence of highest porosity, superior cell viability outcomes, and the most interconnected pore geometry collectively justified the selection of the Tri-Hexagon pattern for drug-loaded graft fabrication. Consequently, Tri-Hexagon fill pattern was selected to produce drug-loaded 3D-printed grafts and the related characterization analysis. Following the selection of the printing pattern, models were printed using drug-loaded composite filaments, and these 3D-printed structures underwent cell toxicity evaluations.

### 3.2 Characterization of 3D-Printed Drug-Loaded Grafts

#### 3.2.1 Morphological Characteristics

The surface morphologies of printed Tri-Hexagon grafts loaded with drugs are shown in Figure 4.

**Figure 4:**
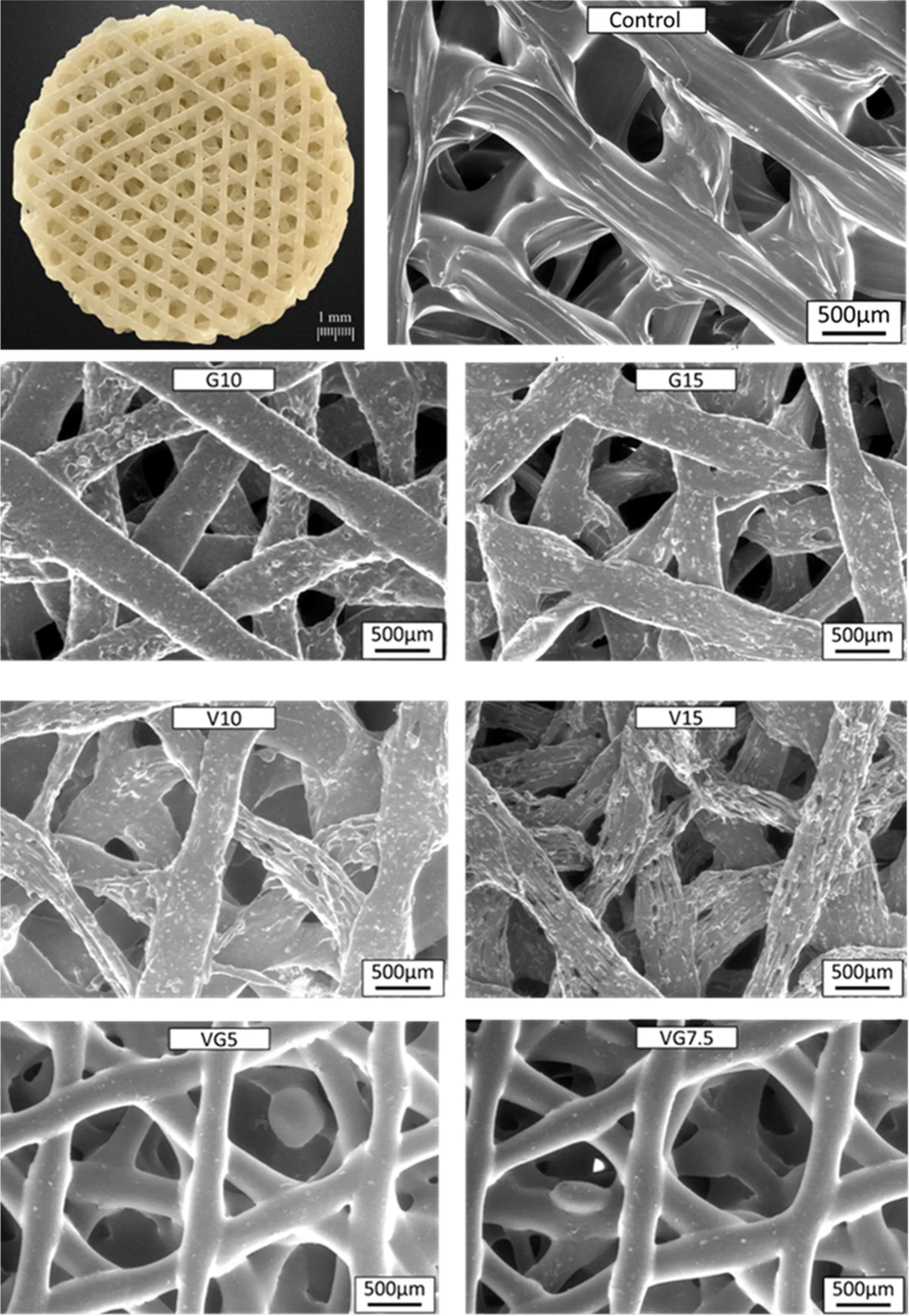
SEM images of 3D-printed and drug-loaded model grafts.

SEM analysis of the optimized Tri-Hexagon scaffolds demonstrated that the 3D printing process effectively accommodated high antibiotic loading without compromising the model structure. Grafts printed without drugs showed relatively smooth surfaces, whereas drug-loaded grafts had rougher surfaces, depending on the incorporated drug type and its concentration. The transition from a smooth control surface to a granular, particulate-laden morphology in the G-, V-, and VG-loaded grafts suggested that the antibiotics were successfully integrated within the PCL/HA matrix, creating a microrough topography favorable to bone cell attachment while maintaining the open-pore network required for nutrient transport. The observed rougher surfaces might be attributed to the increased stickiness and reduced flowability of the filament due to the molten state of the drug.^32^

#### 3.2.2 Antimicrobial Activity

The antimicrobial activities of drug-loaded composite filaments were assessed against *S. aureus* (ATCC 25923), accounting for approximately 50% of osteomyelitis cases, as the representative Gram-positive pathogen, and *E. coli* (ATCC 25922) as the representative Gram-negative organism to provide a broad-spectrum evaluation of the dual-antibiotic system. It is acknowledged that *P. aeruginosa* and methicillin-resistant *S. aureus* (MRSA) represent clinically more prevalent Gram-negative and drug-resistant pathogens in osteomyelitis, respectively; their exclusion was due to biosafety laboratory constraints at the study site and constitutes a limitation to be addressed in future work. The antimicrobial efficacy of grafts is shown in Table 2.

**Table 2:** Antimicrobial Effectiveness of 3D-Printed Drug-Loaded Model Grafts^a^

	inhibition zone (cm)		logarithmic reduction (log CFU/mL)	
sample	*E. coli*	*S. aureus*	*E. coli*	*S. aureus*
control	-	-	-	-
V10	1.0 ± 0.3^b^	0.9 ± 0.2^c^	2.87 ± 0.01^c^	2.21 ± 0.09^c^
V15	2.9 ± 0.2^a^	3.0 ± 0.2^ab^	*^a^	*^a^
G10	0.9 ± 0.2^b^	0.8 ± 0.1^c^	5.58 ± 0.01^b^	2.77 ± 0.03^b^
G15	2.9 ± 0.1^a^	3.0 ± 0.1^ab^	*^a^	*^a^
VG5	2.9 ± 0.1^a^	2.8 ± 0.2^b^	2.10 ± 0.03d	*^a^
VG7.5	3.1 ± 0.1^a^	3.2 ± 0.1^a^	*^a^	*^a^

a Results indicated with different letters in the same column are statistically different according to Tukey’s multiple comparison test (*p* < 0.05). *Completely inhibited.

Specifically, at the highest single-antibiotic loading, V15 and G15 formulations produced inhibition zones of 3.0 ± 0.2 cm and 3.0 ± 0.1 cm against *S. aureus* and 2.9 ± 0.2 cm and 2.9 ± 0.1 cm against *E. coli*, respectively. The dual-antibiotic VG7.5 formulation produced the largest inhibition zones overall (3.2 ± 0.1 cm against *S. aureus*; 3.1 ± 0.1 cm against *E. coli*), achieving full bacterial suppression in both broth and agar assays at a lower total antibiotic burden than the single-antibiotic 15% formulations. V10 and G10 grafts containing a single drug at a 10% ratio had limited inhibition activity against both *E. coli* and *S. aureus* pathogens, while V15 and G15 grafts (15% antibiotic) completely inhibited both pathogens. The VG5 and VG7.5 grafts, combining both drugs, demonstrated effective bacterial inhibition at all tested concentrations. Figure 5 shows representative images of antimicrobial efficacy of drug-loaded model grafts against *E. coli* and *S. aureus* in broth and agar mediums.

**Figure 5:**
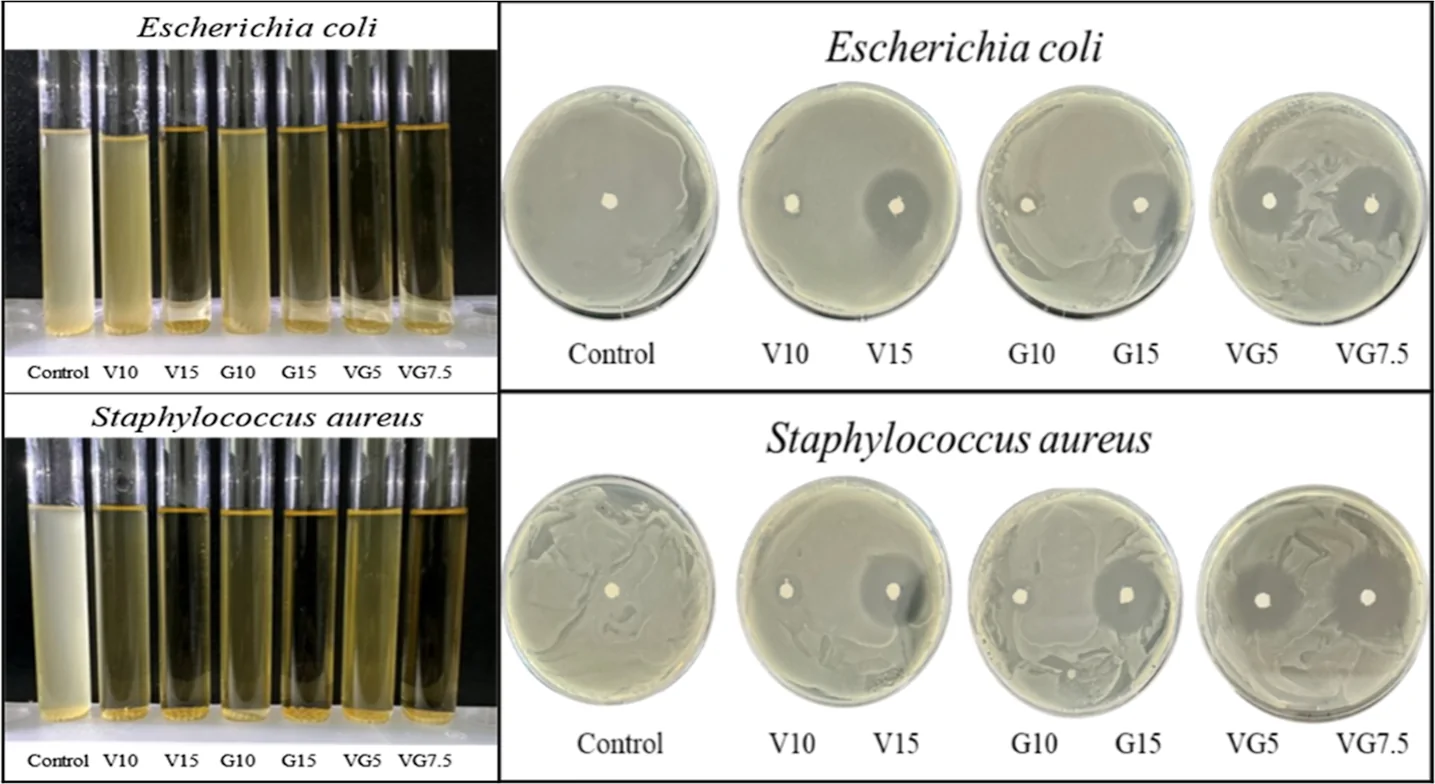
Antimicrobial efficacy of 3D-printed drug-loaded model grafts against *E. coli* and *S. aureus*. Photograph courtesy of O. Sogut. Copyright 2026.

A formal synergy determination was not performed in the present study; however, the antibacterial data for the combined VG formulations were consistent with an additive effect. VG5 (5% vancomycin +5% gentamicin, total antibiotic content 10%) achieved complete inhibition of both *S. aureus* and *E. coli* in the liquid culture assay, a performance level equivalent to the single-antibiotic V15 and G15 formulations containing 15% antibiotic loading, suggesting that the codelivery of vancomycin and gentamicin might enable comparable broad-spectrum antibacterial efficacy at a substantially lower total antibiotic burden. Formal determination of the interaction between these two antibiotics was beyond the scope of this investigation.

#### 3.2.3 Release Behavior of 3D-Printed Drug-Loaded Model Grafts

Cumulative drug release was calculated as the percentage of drug released relative to the total theoretical drug content of each graft, determined as the product of the measured graft weight and the nominal drug-loading fraction (w/w) of the respective filament formulation (Table 3). The validity of this theoretical drug content assumption was further reinforced by FTIR and Raman spectroscopic mapping of the composite filaments (Figure S8), which confirmed a spatially homogeneous distribution of both vancomycin and gentamicin throughout the PCL/HA matrix without observable phase separation, preferential surface localization, or drug aggregation, indicating that the nominal loading fraction reliably represented the bulk drug content of the composite material. Similarly, isothermal TGA at 200 °C under air conditions demonstrated that both vancomycin and gentamicin retained thermal stability under the extrusion conditions applied (Figure S12); and the extrusion temperature (170–180 °C) remained below the onset degradation temperatures recorded for all formulations (Table S4).

**Table 3:** Cumulative Release Amount of Model Grafts after 21 Days

sample	mass (mg)	drug content (mg)	released amount (mg)	cumulative release (%)
V10	984	98.4	29.54	30.02
V15	978	146.7	38.17	26.01
G10	975	97.5	65.18	66.18
G15	854	128.1	79.26	61.87
VG5	945	94.5	46.16	48.84
VG7.5	938	140.7	56.77	40.34

Tandem mass spectrometry (LC-MS/MS) was used to quantify the postrelease concentrations of vancomycin and gentamicin with high sensitivity and specificity. Initial infusion scans were performed to characterize the parent and fragment ions for both antibiotics. For vancomycin, the parent ion was identified at *m*/*z* 1450. Collision-induced dissociation at 45 V generated characteristic fragment ions at *m*/*z* 1088.96, 1117.74, 1145.5, 1279.56, 1307.59, and 1383.33. The ions at *m*/*z* 1145.5 and 1307.8 were selected as the quantifier and qualifier transitions for the monitoring of vancomycin (Figure 6).

**Figure 6:**
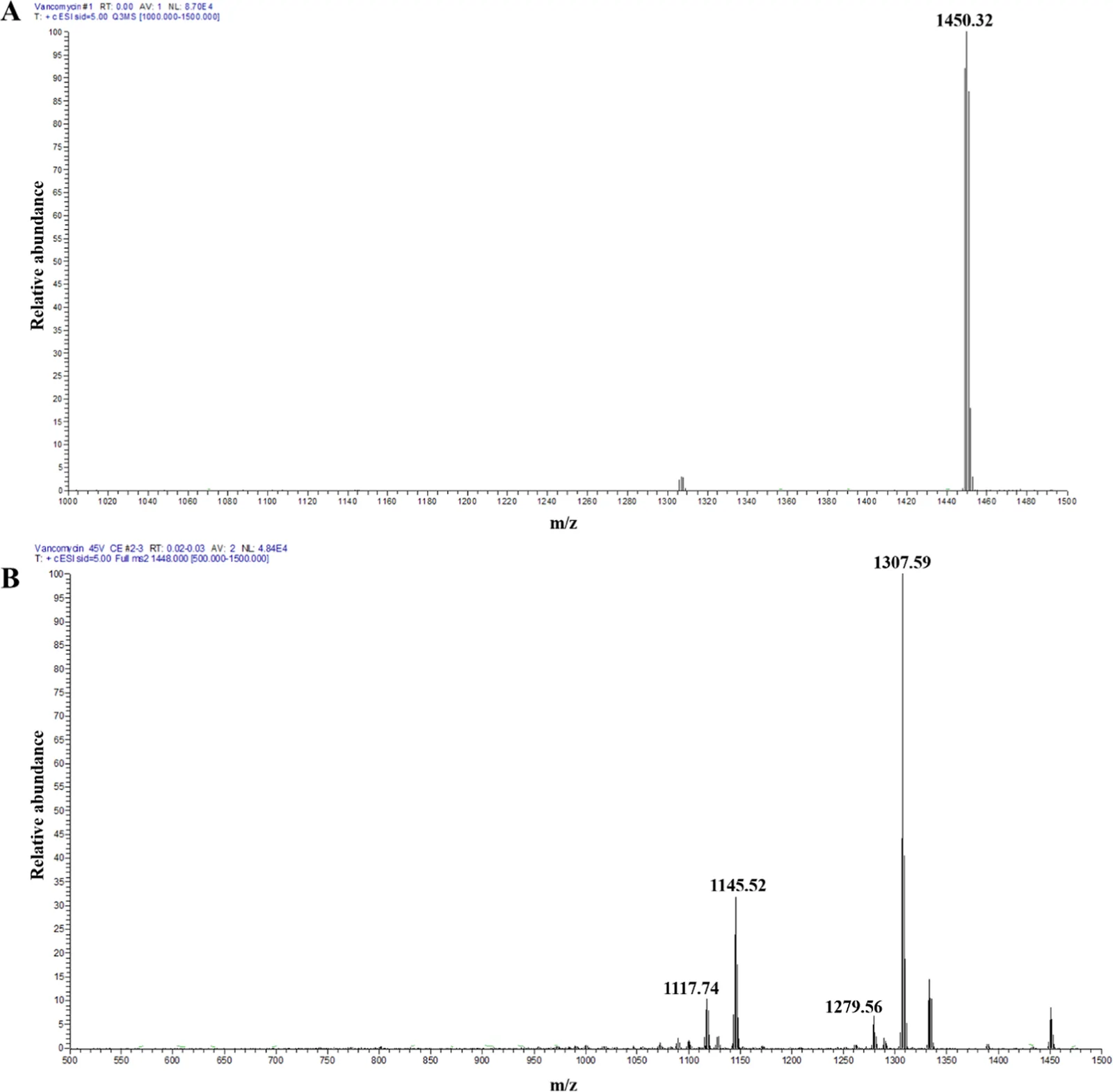
postinfusion parent ion mass spectrum (A) and the fragmentation ion spectrum of the 1450 Da parent ion (B) of vancomycin.

In contrast, gentamicin had multiple parent ions corresponding to its natural isoforms, gentamicin C1 (*m*/*z* 478.2), C1a (*m*/*z* 450.3), and C2 (*m*/*z* 464.3) (Figure 7). The total gentamicin concentration was reported as the sum of these three components. Literature research indicated that these ions correspond to Gentamicin’s natural components, denoted as Gentamicin C1, Gentamicin C1a, and Gentamicin C2.^33^ The parent ion mass of Gentamicin C1a was determined as 450.3 Da, yielding the most significant fragmentation ions at 160.1 and 322.2 Da at 15 V collision energy. Similar fragmentation patterns were observed for Gentamicin C1a and C2 at collision energies of 15 and 12 V, respectively.

**Figure 7:**
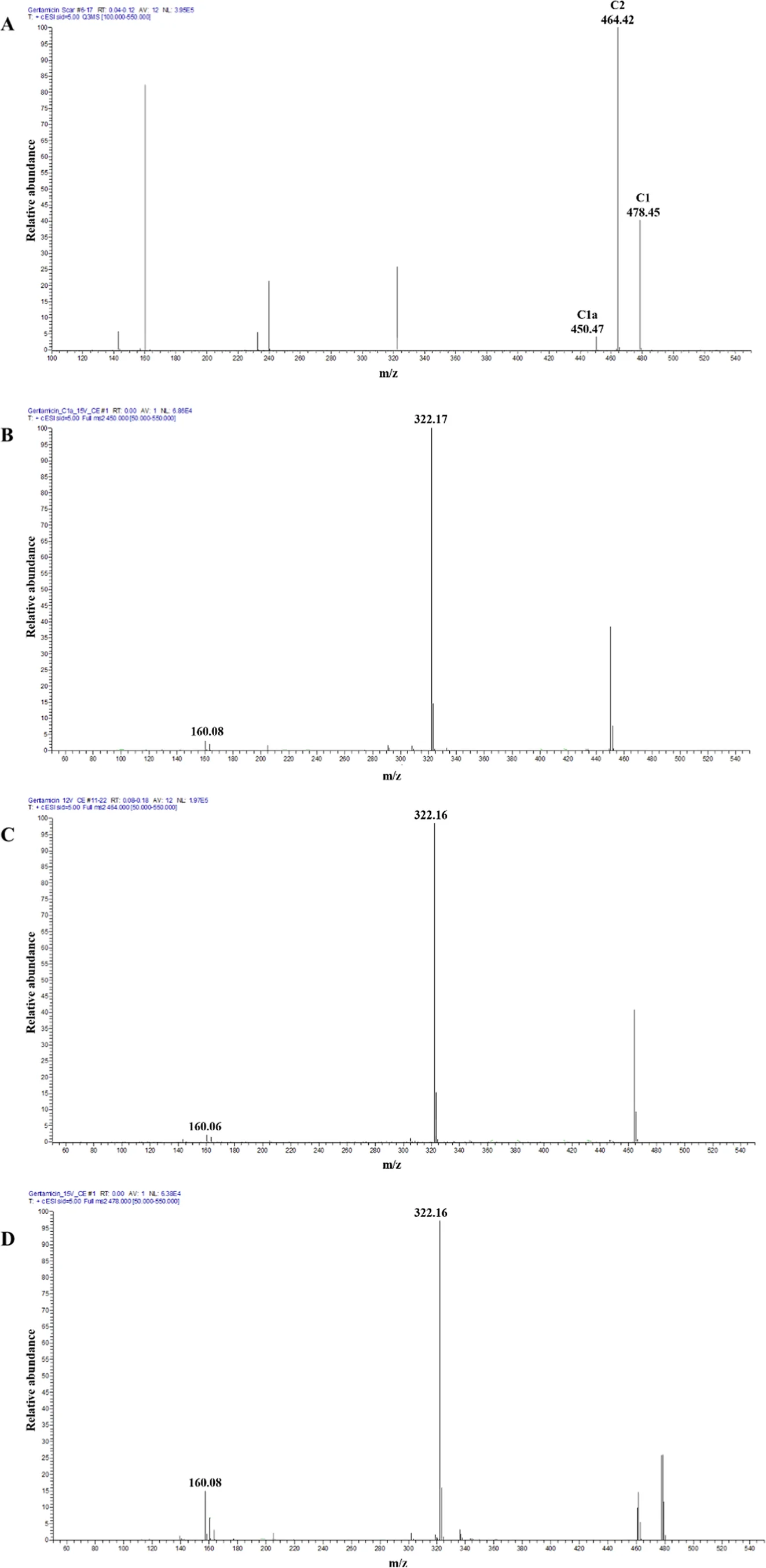
Postinfusion parent ion mass spectrum of gentamicin (A), the fragmentation ion spectrum of the 450 Da parent ion of gentamicin C1a (B), the 464.00 Da parent ion of gentamicin (C), and the 478.00 Da parent ion of gentamicin (D).

The fragmentation patterns of both drugs are shown in Table 4, where MRM quantifier/qualifier transitions, cone voltage, dwell time, retention times, limit of detection, limit of quantitation, and full method validation parameters (accuracy, precision, and matrix effect) per ICH Q2­(R1) are provided in Supporting Information Table S7.

**Table 4:** Parent Masses and Fragmentation Ions of Vancomycin and Gentamicin Determined by Infusion-Based Tandem Mass Spectrometry

compound	main ion (*m*/*z*)	fragmentation compound (*m*/*z*)	collision energy (V)
gentamicin C1a	450.3	160.1	15
	450.3	322.2	15
gentamicin C2	464.3	160.1	12
	464.3	322.2	12
gentamicin C1	478.2	157.1	15
	478.2	322.2	15
	478.2	461.6	15
vancomycin	1450.8	1145.5	45
	1450.8	1307.8	45

The released drug amounts from drug-loaded model grafts were determined using mass spectrometric data obtained periodically using tandem mass spectrometry. For the standard curves, standard mixtures containing 10, 20, 50, and 100 μg/mL of vancomycin and gentamicin were prepared, and calibration curves were constructed for quantification. Polynomial regression equations of the second degree were applied to these curves, resulting in *R* ^2^ values exceeding 0.999 for each equation.

The release profiles of drugs from the model grafts are shown in Figure 8.

**Figure 8:**
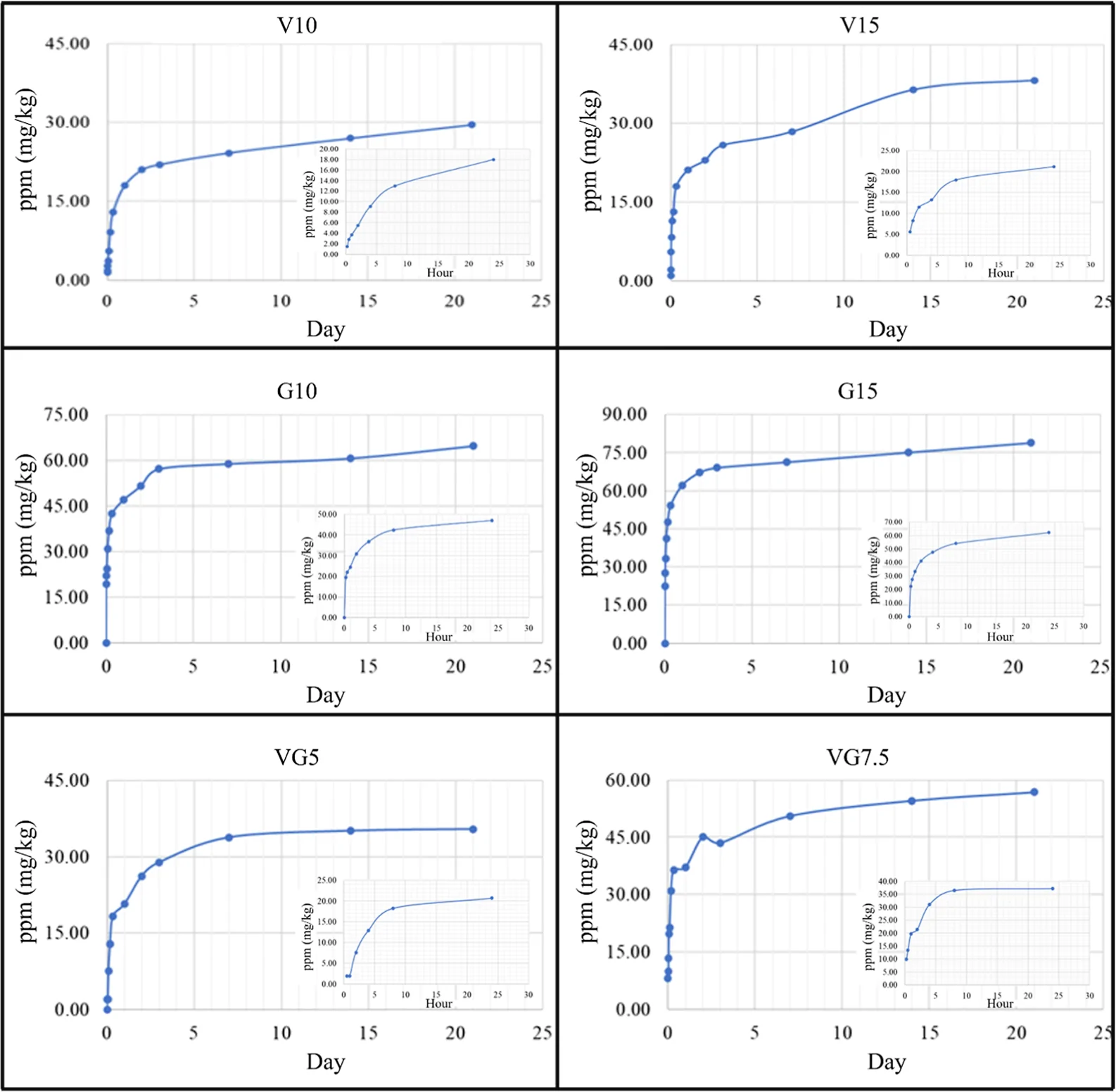
Release profiles of vancomycin from V10 and V15 grafts and of gentamicin from G10 and G15 grafts.

The release profiles of all 3D-printed composite grafts had a characteristic biphasic pattern where a rapid burst release occurred within the first 72 h followed by a prolonged, sustained release phase. Over the 21 day experiment, gentamicin-loaded grafts reached significantly higher cumulative release (∼62–66%) compared to vancomycin-loaded counterparts (∼26–30%). This difference might be related to the physicochemical properties of the drugs. Gentamicin, a highly hydrophilic aminoglycoside (*M*_W_ ∼ 450 Da; aminoglycoside; highly hydrophilic; log *P* – 1.8), could diffuse more readily through the hydrophobic PCL matrix than the larger, amphiphilic vancomycin (*M*_W_ ∼ 1450 Da; glycopeptide; amphiphilic). This behavior was found to be consistent with the inverse relationship between molecular weight and the diffusion coefficient, as described by the Stokes–Einstein equation,^34^ and the semicrystalline and hydrophobic nature of PCL matrix.^35^ Besides, over 21 days, the VG5 graft achieved a cumulative release of 48.84% and the VG7.5 graft released 40.34%. The addition of drugs as a combination indicated that the presence of both drugs had an intermediate elution profile, potentially offering a broader spectrum of antibacterial coverage while balancing release rates.

To evaluate the release mechanism, the data were fitted to various kinetic models. A comparative model selection table (*r* ^2^, AIC) among candidate models tested (zero-order, first-order, Higuchi, and logarithmic) is provided in Table S8. The logarithmic empirical model (*y* = *a*·ln­(*x*) + *b*) provided the highest correlation (*r* ^2^ > 0.988) for all formulations. Furthermore, the Korsmeyer–Peppas model was applied to the early release data within the *M*t/*M*∞ ≤ 0.60 range (i.e., data points where cumulative fractional release did not exceed 60%), consistent with the boundary conditions of the power-law model. The calculated release exponent n ranged from 0.395 to 0.435 across all formulations (G10: *n* = 0.424; G15: *n* = 0.435; V10: *n* = 0.414; V15: *n* = 0.410; VG5: *n* = 0.399; and VG7.5: *n* = 0.395), all below *n* = 0.45, confirming Fickian diffusion as the dominant release mechanism across all antibiotic types and loading concentrations.

The Fickian diffusion-dominated release observed in this study could be mechanistically attributed to two concurrent factors. First, the hydrophilic HA nanoparticles embedded within the hydrophobic PCL matrix might form interconnected aqueous diffusion microchannels upon immersion in PBS, providing preferential pathways for drug molecules to migrate toward the release medium. Second, the marked difference in cumulative release between gentamicin (∼62–66%) and vancomycin (∼26–30%) might be consistent with the inverse relationship between molecular weight and the Fickian diffusion coefficient: gentamicin (*M*_W_ ∼ 450 Da, log *P* – 1.8) possesses substantially higher aqueous diffusivity than vancomycin (*M*_W_ ∼ 1450 Da, amphiphilic glycopeptide), enabling faster and more extensive transport through these hydrophilic diffusion channels. The contribution of PCL matrix erosion to overall drug release within the 21 day observation window can be considered negligible, as PCL undergoes hydrolytic degradation on a time scale of months to years under physiological conditions.

Based on the logarithmic empirical model fit and as model-based extrapolations that require further experimental validation, the projected cumulative release after 6 and 12 months was estimated as follows: the V10 filament model would release 37.99% and 40.73%, respectively, while the V15 filament model would release 31.08% and 33.22%, respectively. Based on the fitted equations, the G10 and G15 filament models were projected to release approximately 78.83% and 76.22%, respectively, after 6 months, and 83.11% and 80.26%, respectively, after 12 months. Model-based extrapolations also projected that VG5 would release 58.10% and 62.53%, while the VG7.5 model would release 47.85% and 50.92% after 6 and 12 months, respectively. It must be explicitly emphasized that these projections were model-based mathematical extrapolations derived from 21 day in vitro release data and must not be interpreted as validated evidence of long-term drug release. The logarithmic empirical model cannot account for PCL matrix hydrolytic degradation, changes in local pH, protein adsorption, or the dynamic mechanical and biological environment present in vivo, all of which would substantially influence actual release behavior beyond the experimental window. These estimates can be provided solely as a theoretical reference to indicate the potential for extended-release kinetics under idealized conditions, with experimental validation at time points of ≥3 months under both in vitro and in vivo conditions being required before any conclusions regarding long-term therapeutic efficacy can be supported.

#### 3.2.4 Cytotoxicity

The cell viability results of drug-loaded grafts via a multiple-regression graph, enabling a thorough comparison of viability trends among various experimental groups, are shown in Figure 9.

**Figure 9:**
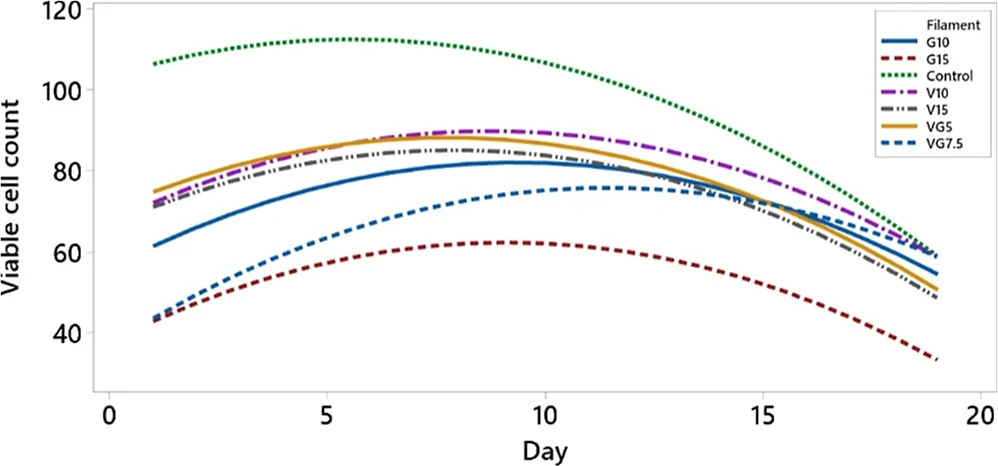
Viable cell counts of grafts found in in vitro cytotoxicity studies.

Cytotoxicity assays demonstrated that all grafts had good biocompatibility, and the integration of gentamicin and vancomycin through the 3D printing process did not compromise the safety of the grafts, suggesting a generally acceptable safety profile in the context of localized drug delivery for osteomyelitis, pending further formulation optimization. Initial lower cell viabilities were attributed to high local drug concentrations exceeding the cytotoxic threshold for osteoblast-like cells during the burst release phase, consistent with the known concentration-dependent cytotoxicity of aminoglycosides and glycopeptides at supraphysiological local doses.^36,37^ The pH of the culture medium remained stable across all grafts (7.29–7.42), which did not impair cellular adhesion, proliferation, and the synthesis of the extracellular matrix. Despite these initial effects, cell viability levels in subsequent phases reached comparable levels among most grafts, except G15 grafts. At the end of the study, the VG7.5 and V10 groups showed similar live cell counts to the control group, followed by G10, VG5, and V15. These results suggested that higher concentrations of drugs than the chosen concentrations might not support cell survival. From a clinical safety perspective, the substantially reduced osteoblast viability observed with G15 at day 21 (66% lower than control) warrants careful consideration: while local aminoglycoside concentrations far exceeding systemic minimum inhibitory concentration (MIC) values are commonly used in clinical bone infection management to achieve bactericidal local levels, the cytotoxic threshold for osteoblast-like cells in vitro must be balanced against antibacterial efficacy in future formulation optimization. The G15 formulation should, therefore, not be advanced to further preclinical studies without dose adjustment or matrix modification to attenuate the sustained high local gentamicin concentration. It should also be noted that the cytotoxicity assay (ISO 10993-5 compliant) performed in this study represents an initial, required screening step within the broader ISO 10993-1 biological evaluation framework for medical devices. A comprehensive biocompatibility assessment for regulatory submission would additionally require genotoxicity, sensitization, and systemic toxicity testing, which are planned as part of future preclinical development.

As a prespecified sensitivity analysis to assess long-term viability independent of the burst-release cytotoxic phase, statistical analyses were repeated excluding the first week of culture. The control group and models produced with V10 filaments did not exhibit statistically significant differences in terms of viability (*p* < 0.05). Similarly, the V10, VG5, VG7.5, G10, and V15 groups showed no significant differences in cell viability (*p* < 0.05). However, the G15 graft had the lowest viability compared with all other grafts, where it had 39% fewer live cells than the subsequent V15 graft and 66% fewer live cells than the control group, as shown in Table 5.

**Table 5:** Cell Viability of Drug-Loaded Grafts after 21 Days^a^

sample	*N*	cell count
control	20	88.59 ± 17.02^a^
V10	20	80.79 ± 16.10^ab^
VG5	20	77.29 ± 20.38^b^
VG7.5	20	74.77 ± 16.59^b^
G10	20	74.67 ± 13.65^b^
V15	20	73.95 ± 18.00^b^
G15	20	53.12 ± 16.66^c^

a Results indicated with different letters in the same column are statistically different according to Tukey’s multiple comparison test (*p* < 0.05).

#### 3.2.5 Mechanical Properties

The compressive mechanical properties of 3D-printed grafts are summarized in Table 6, and representative stress–strain curves are presented in Figure 10.

**Table 6:** Compressive Mechanical Properties of 3D-Printed Drug-Loaded Grafts^a^

sample	compressive modulus (MPa)	max stress (MPa)
control	7.99 ± 1.39^a^	106.35 ± 7.38^ab^
V10	7.88 ± 3.13^a^	105.76 ± 8.21^b^
V15	7.77 ± 3.29^a^	102.53 ± 8.12^b^
G10	7.58 ± 1.04^a^	102.94 ± 6.79^a^
G15	7.44 ± 0.57^a^	95.95 ± 7.30^ab^
VG5	7.52 ± 1.86^a^	101.28 ± 7.50^ab^
VG7.5	7.47 ± 2.43^a^	100.94 ± 9.02^ab^

a Results indicated with different letters in the same column are statistically different according to Tukey’s multiple comparison test (*p* < 0.05).

**Figure 10:**
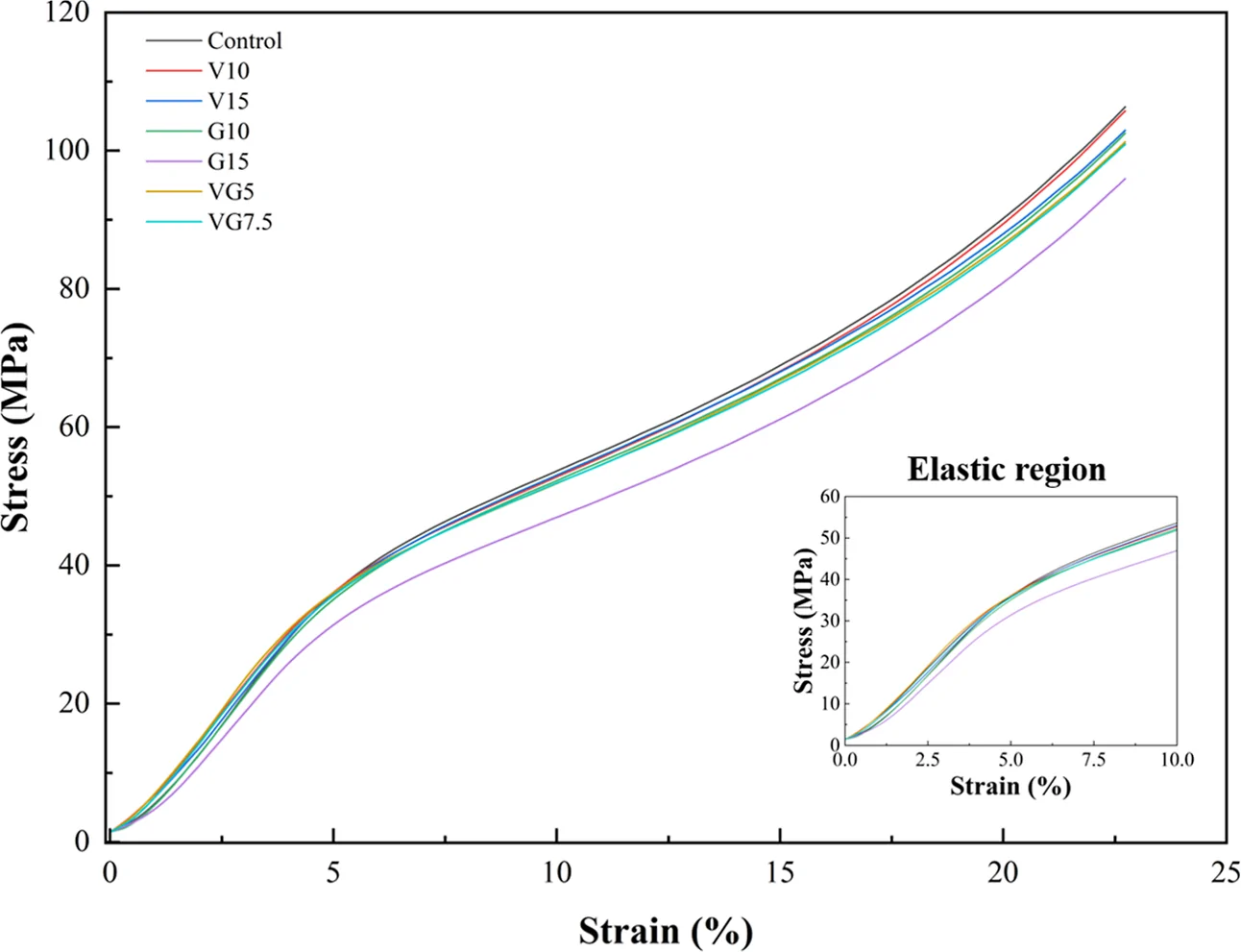
Stress–Strain Curve graph of 3D-printed models.

All groups exhibited the characteristic three-stage deformation profile of porous polymeric grafts under compression: a linear elastic region (0–3% strain) governed by cell wall bending and strut deformation, a collapse plateau (3–20% strain) associated with progressive pore strut buckling and failure, and a densification region beyond approximately 20% strain where opposing struts make contact and the stress rises steeply.

The compressive strength of the antibiotic-free control PCL/HA graft was 7.99 ± 1.39 MPa, which is comparable to cancellous bone (2–45 MPa) and consistent with previous studies on 3D-printed PCL/HA scaffolds.^38,39^ The compressive modulus ranged from 7.44 MPa (G15) to 7.99 MPa (Control), where the rank order of modulus was Control > V10 > V15 > G10 > VG5 > VG7.5 ≈ G15. No statistically significant differences were detected between any of the groups for either compressive modulus (*p* > 0.05) or yield strength (*p* > 0.05). The strain at maximum stress was also consistent across all groups at 22.73%, confirming that the densification onset was unaffected by drug type or concentration. The vancomycin-loaded groups showed modest decreases in modulus relative to Control, while increasing vancomycin to 15% resulted in slightly lower values compared to V10. The gentamicin-loaded groups showed the lowest values across all metrics, while dual-drug groups (VG5 and VG7.5) produced better modulus values. The incorporation of vancomycin and/or gentamicin at 5–15% concentrations did not significantly alter the compressive mechanical properties of the PCL/HA scaffold system, confirming that clinically relevant doses of these antibiotics could be incorporated without compromising structural integrity. The consistent deformation behavior across all groups, identical three-stage profile, and consistent densification onset at approximately 22.7% strain, further supported this conclusion.

## 4 Discussion

Tissue engineering is a rapidly developing discipline that combines several components, including scaffolds, stem cells, and bioactive substances, to enable tissue repair and regeneration. Thus, the present work evaluated the addition of antibiotics into 3D-printed grafts intended to be used for osteomyelitis treatment by emphasizing their physicochemical characteristics, antibacterial action, and biocompatibility. The proposed approach aimed to improve therapeutic results by encouraging bone tissue regeneration simultaneously with controlled medication release at the infection site, thereby enabling localized antibiotic delivery. PCL has been mostly used in studies due to its low melting temperature (∼60 °C), appropriate rheological features for melt extrusion, and better biocompatibility.^40,41^ The addition of nano-HA (nHA) has been particularly significant, as it would enhance osteoconductivity, a critical point for promoting cell adhesion and osteogenic differentiation.^42,43^ A fundamental first step in bone tissue engineering has been documented as cell adhesion; thus, the presence of nHA was found to improve this process. For example, Song et al. found that the increased hydrophilicity and availability of additional adhesion sites on the scaffold surface of nHA greatly enhanced cell attachment when included in PCL scaffolds.^44^ Similar to that, Hokmabad et al. underlined that improved adherence of osteoblasts, which is essential for later proliferation, is made possible by the nanorough surface produced by nHA particles.^45^ This trend is consistent with the results of Sattary et al., who observed that nHA-containing scaffolds showed higher alkaline phosphatase (ALP) activity, therefore, reflecting improved osteogenic differentiation.^46^ The results of the study on the morphology and microstructure of the grafts correspond with earlier studies stressing the significance of polymer chain entanglement during extrusion, which maintains improved mechanical stability and fiber alignment, thus ensuring better load distribution inside the scaffold.^47^

SEM images of produced grafts confirmed that the 3D-printed grafts had pores of varying sizes, ranging from 200 to 1500 μm, and showed interconnected voids that could promote cell adhesion, nutrient exchange, and waste removal,^48^ which was consistent with findings from other studies that suggested a pore size greater than 300 μm was optimal for enhancing bone defect filling.^49^ This is corroborated by Wu et al., who demonstrated that scaffolds with pore sizes ranging from 300 to 500 μm facilitated tissue ingrowth and nutrient supply.^50^ Furthermore, the porosity of the current grafts was designed to be high, which was crucial for cell migration and vascularization, aligning with the findings of Montelongo et al., who optimized scaffold architecture to achieve increased porosity while maintaining mechanical strength.^51^

Proliferation rates on PCL/nHA scaffolds have also been shown to be superior compared with pure PCL scaffolds. For example, Chuenjitkuntaworn et al. demonstrated that cells cultured on PCL/nHA scaffolds exhibited higher proliferation rates, particularly after the initial 10 day culture period, suggesting that nHA provided a conducive environment for cell growth.^52^ Furthermore, Kiran et al. found that the addition of collagen to PCL/nHA scaffolds significantly enhanced cell attachment and proliferation, indicating that the combination of materials can synergistically improve cell behavior.^53^ FTIR and XRD results for the produced grafts confirmed the stability of the drug-loaded grafts, indicating successful incorporation of antibiotics without compromising the material properties, a finding consistent with other studies that have explored the integration of bioactive compounds into scaffolds.^54^

The antimicrobial activity of the antibiotic-loaded grafts against *S. aureus* and *E. coli* demonstrated a notable combinatorial antibacterial effect at the tested concentrations, particularly with the gentamicin-vancomycin combination. It should be noted that the current study reports antibacterial activity in terms of zone of inhibition diameters and log CFU/mL reductions and does not include MIC determination against the tested strains. Accordingly, the released drug concentrations relative to established MIC breakpoints (EUCAST/CLSI) remain to be characterized in future work, which represents a limitation in directly inferring the clinical therapeutic equivalence of the observed in vitro inhibition. In the current study, the release profile of gentamicin and vancomycin exhibited an initial burst followed by a sustained release phase. This observed release profile might effectively prevent early stage infection while promoting long-term implant stabilization, corroborating findings from other studies that have examined similar drug delivery systems in tissue engineering.^55,56^ The release characteristics of these antibiotics are critical for ensuring effective local treatment of infections, especially in the context of osteomyelitis.^57^ The initial burst is typically associated with the rapid diffusion of drugs located near the surface of the scaffold, while the sustained release phase is governed primarily by Fickian diffusion through the polymer matrix, as supported by the Korsmeyer–Peppas exponent values (*n* = 0.395–0.435) obtained in this study. While polymer erosion may contribute incrementally over longer timeframes, the dominant mechanism within the 21 day observation window is diffusion-controlled. From a mechanistic standpoint, the differential release behavior between gentamicin and vancomycin in this study required further interpretation. The substantially higher cumulative release of gentamicin (∼62–66%) compared with vancomycin (∼26–30%) over 21 days might be primarily attributable to the marked differences in the physicochemical properties of these two antibiotics. Gentamicin, a low-molecular-weight aminoglycoside (∼450 Da) with high hydrophilicity (log *P*-1.8), might facilitate rapid partitioning into the aqueous PBS medium and fast diffusion through the hydrophilic microchannels formed by the HA nanoparticle network embedded within the semicrystalline PCL matrix. In contrast, vancomycin, a bulky glycopeptide (∼1450 Da) with amphiphilic character, whose significantly higher molecular weight might result in a substantially lower diffusion coefficient as predicted by the Stokes–Einstein relationship and whose partial hydrophobic character might promote noncovalent interactions with the PCL backbone, further retarding its release. The present PCL/HA system has potential over established local antibiotic delivery platforms: unlike nonbiodegradable PMMA bone cement beads, which require a second surgical procedure for retrieval, PCL can undergo gradual hydrolytic degradation under physiological conditions, offering the prospect of a single-stage surgical intervention. Furthermore, compared with rapidly resorbing calcium sulfate carriers (e.g., Cerament G, Stimulan), which are fully resorbed within weeks, the slower degradation profile of PCL may better support the prolonged drug exposure required for the eradication of chronic osteomyelitis. However, these comparative advantages remain theoretical in the absence of direct in vivo comparative data, which represents an essential step in the translational development of the present system.

In contrast to the Fickian-dominant mechanism observed in this study, attributed to the hydrophilic HA nanoparticle network creating diffusion pathways within the PCL matrix, Jiang et al. demonstrated that the release of drugs from pure PCL matrices was primarily controlled by erosion, with a release exponent (*n*) indicating a near-linear profile, suggesting that the drug release was predominantly erosion-based.^58^ Yuan et al. also noted that drug-loaded fiber mats could exhibit various release mechanisms, including diffusion and polymer degradation.^59^ This pattern is consistent with findings from other studies. For instance, Li et al. reported that gentamicin-loaded bone cement demonstrated a similar initial burst release, with only about 4% of gentamicin eluted after a certain period.^60^ This initial burst is often attributed to the rapid diffusion of the drug from the surface of the scaffold or cement, which is a common characteristic observed in various antibiotic delivery systems.^61^ Moreover, Slane et al. highlighted that high doses of antibiotics, such as tobramycin and vancomycin, incorporated into acrylic bone cement also exhibited a significant initial release followed by a gradual decrease in elution rates over time.^62^ This supports the notion that the initial burst release is a common phenomenon across different delivery systems, which can be beneficial for quickly achieving therapeutic concentrations at the infection site. The sustained release phase observed in the current study is particularly noteworthy. Li et al. found that the sustained release of vancomycin and gentamicin from acrylic bone cement was limited, with only 2.33–6.16% of vancomycin and 3.39–3.99% of gentamicin released over a longer duration.^60^ In contrast, the grafts developed in the current study may provide a more favorable sustained release profile; however, direct cross-system comparisons are limited by differences in scaffold material, geometry, and release medium conditions, potentially due to the unique 3D-printed architecture that allows for better control over the release kinetics. This study’s results align with the existing literature on antibiotic-loaded scaffolds for osteomyelitis treatment, further emphasizing the potential of 3D-printed personalized scaffolds in regenerative medicine.

Compared with clinically established antibiotic delivery systems such as calcium sulfate-based carriers (Cerament G, Stimulan) and PMMA bone cement beads, the 3D-printed PCL-HA grafts offer biodegradability and a patient-adaptable design concept as key advantages. These attributes are strongly supported by the latest studies, which highlighted that the localized codelivery of vancomycin and gentamicin via 3D-printed architectures provided better eradication of osteomyelitis-associated biofilms compared to traditional single-antibiotic bone cements while maintaining necessary structural integrity.^63,64^ However, direct performance comparisons with these established products remain limited in the absence of equivalent in vivo experimental data. Although this study is an initial framework for 3D-printed antibiotic-loaded bone grafts, further research is required to enhance its translational potential. Future investigations should focus on in vivo validation using large animal models, such as sheep or horses, to better simulate human physiological responses and overcome the inherent limitations of rodent models in osteomyelitis research. A further limitation of the present study is that antimicrobial evaluation was confined to reference laboratory strains (*S. aureus* and *E. coli*) under planktonic growth conditions. Testing against clinically relevant drug-resistant pathogens, particularly methicillin-resistant *S. aureus* (MRSA) and biofilm-forming clinical isolates, is essential for a comprehensive assessment of the system’s therapeutic utility in real-world osteomyelitis scenarios. Longitudinal studies exceeding the 21 day observation window are also necessary to fully characterize the long-term degradation kinetics and mass loss of the PCL matrix under physiological conditions. While the geometrical framework and 30°layer rotation strategy applied in the present study were shown to yield high porosity and favorable cell compatibility for PCL-based scaffolds in a preceding study from our group,^29^ the compressive strength and elastic modulus of drug-loaded PCL/HA grafts showed that antibiotic incorporation into grafts did not compromise the structural integrity or load-bearing capacity required for orthopedic applications in their final drug-loaded state. The compressive mechanical properties of the present 3D-printed PCL/HA grafts (35.91–39.95 MPa compressive strength; 729.85–852.52 MPa elastic modulus) were within the range of values reported for cancellous bone, which typically exhibited compressive strengths of 2–45 MPa and elastic moduli of 0.1–5 GPa.^65,66^ The retention of mechanical properties upon antibiotic incorporation observed in the present study is consistent with previous investigations in which drug incorporation into polymeric melt-extruded matrices was reported to increase surface roughness without substantially affecting bulk mechanical integrity.^67,68^ It should be noted that mechanical testing in the present study was conducted under static, dry conditions; the response of PCL-based grafts to dynamic, cyclic, and hydrated loading was not assessed and constitutes a recognized limitation of the current investigation. The cell viability and cytotoxicity assays performed (ISO 10993-5 compliant) represented the minimum required biological characterization for an antimicrobial drug delivery device at the proof-of-concept stage. However, further studies including osteogenic differentiation capacity, ALP activity, calcium deposition, or the expression of osteogenic markers such as RUNX2, COL1A1, or osteocalcin are required to evaluate localized antibiotic delivery for infection control rather than bone regeneration.

## 5 Conclusion

This study evaluated the significant potential of 3D printing in advancing osteomyelitis treatment through the development of antibiotic-loaded composite grafts. The combination of PCL and HA in the graft production step showed both the adaptability of 3D printing to intricate biomaterial structures and the advantageous properties of the materials. The resultant porous structure within the grafts enhanced the surface area for cell adhesion and nutrient exchange and supported cell viability and tissue regeneration. The incorporation of antibiotics, particularly gentamicin and vancomycin, at varying concentrations demonstrated combinatorial antibacterial activity, effectively inhibiting osteomyelitis-associated reference strains (*S. aureus* ATCC 25923 and *E. coli* ATCC 25922). Furthermore, analyses of physicochemical properties, thermal stability, and drug-release kinetics showed the potential clinical utility of these composite grafts. The controlled initial drug release followed by a sustained pattern obtained in this study could enhance therapeutic efficacy and translational capacity. In summary, the present study provided proof-of-concept in vitro evidence that 3D-printed PCL/HA composite grafts could function as patient-adaptable platforms for localized dual-antibiotic delivery in the management of osteomyelitis. All findings were limited to in vitro conditions using reference bacterial strains and a human osteoblast-like cell model and should not be interpreted as evidence of clinical efficacy or safety. Thus, comprehensive preclinical validation, including in vivo animal studies, MRSA efficacy testing, long-term stability assessments, and mechanical characterization, is required before any claim of clinical translational potential can be made.

## Supplementary Material





## Notes

STL file of the base cylindrical geometry for 3D-printed grafts (ZIP)
Microscope images of PCL particles before and after milling; particle size distribution of HA, vancomycin, and gentamicin; FTIR spectra of raw materials; XRD patterns of PCL and HA; images of produced drug-loaded filaments; AFM images of composite filaments; FTIR spectra of composite filaments; FTIR and Raman spectroscopic mapping, 2D and 3D; XRD patterns of composite filaments; TGA and DTG curves of composite filaments; isothermal TGA curves at 200 °C under air; differential scanning calorimetry curves and second heating cycle of composite filaments; calibration curves for vancomycin and gentamicin quantification; particle size of milled samples; crystallinity degree and crystal size of PCL particles; crystallinity and crystal size of composite filaments; thermal characteristics of composite filaments; thermal properties of composite filaments; 3D printing parameters; LC-MS/MS method validation parameters for gentamicin and vancomycin; and kinetic model fitting parameters for drug release (PDF)
