# Supplementary material for: 3D-Printed Vancomycin- and Gentamicin-Loaded Bone Grafts for Osteomyelitis Treatment: Drug Release and Antibacterial Efficacy
Source: ACS Omega. 2026 Aug 3;11(32):48344–60. doi: 10.1021/acsomega.6c04993 (PMC13491886; doi:10.1021/acsomega.6c04993)
Supplement: Supplementary file 1 [file ao6c04993_si_002.pdf]

SUPPLEMENTARY

3D Printed Vancomycin- and Gentamicin-Loaded Bone Grafts for Osteomyelitis Treatment: Drug Release and Antibacterial Efficacy

Oğuz Sogut[1,3], Umran Aydemir Sezer[1,2,*]

[1]Faculty of Medicine, Department of Pharmacology, Medicine, Medical Device and Dermocosmetic Research and Application Laboratory-IDAL, 32260, Isparta, Turkey

[2]Suleyman Demirel University, Institute of Health Sciences, Department of Regenerative Medicine, 32200, Isparta, Turkey

[3]Suleyman Demirel University, Natural Products Application and Research Center, 32200, Isparta, Turkey

*Corresponding author: umransezer@sdu.edu.tr

1. Method

1.1. Characterization of powders

The morphology and size of polycaprolactone (PCL) particles before and after milling were assessed using an inverted microscope (Carl Zeiss, Primovert HDcam, Germany). A particle size analyzer (Horiba, nanoPartica SZ-100V2, Japan) based on dynamic light scattering (DLS) methodology was used for particle size and distribution behavior of hydroxyapatite (HA) and antibiotics obtained after milling. The chemical structures of the raw materials (PCL, nano-HA, vancomycin, and gentamicin) were evaluated using Fourier Transform Infrared (FTIR) spectroscopy (Thermo Scientific, Nicolet iN10, USA). FTIR spectra were collected over a wavenumber range of 4000–400 cm^{-1} at a spectral resolution of 4 cm^{-1}. For each powder sample, 32 co-added scans were recorded to obtain an optimal signal-to-noise ratio. All acquired spectra were baseline-corrected and normalized to the most intense absorption band for comparative analysis. The crystalline structure and phase composition of the PCL and HA powders were evaluated by X-ray diffractometer (XRD) (BRUKER, D8 ADVANCE TWIN-TWIN, Germany) using Cu anode as the X-ray source operated at 40.0 kV and 40.0 mA. The continuous PSD fast technique was used in coupled two theta/theta scanning mode for the measurements. At 25°C, the scan ran from 10° to 80° with a step increase of 0.020°, producing 3570 steps overall.

1.2. Characterization of drug-loaded filaments

1.2.1. Morphological and chemical properties

The surface roughness was measured using an atomic force microscope (NanoMagnetics Instruments, ez-AFM Nanomagnetics, UK). The surface roughness of the composite filaments was measured in a stable state without external interference. The filaments were kept in a 100°C oven for 1 h, and molten filaments were cooled to room temperature for the roughness analysis performed with a scan range of 5×5 μm and a resolution of 256 pixels.

Fourier Transform Infrared (FTIR) spectroscopy (Thermo Scientific, Nicolet iN10, USA) and Raman spectroscopy (Thermo Scientific, DXR3xi Raman Imaging Microscope, USA) were used to detect potential changes after composite fiber production and to determine the distribution of the composite matrix. FTIR spectra were collected over the wavenumber range of 4000–400 cm^{-1} at a spectral resolution of 4 cm^{-1}, with 32 co-added scans per spectrum. Raman spectra were recorded using a 785 nm laser with a laser power of 30 mW in the range of 3500-250 cm^{-1} with a sensitivity of 1.7 μm. Mapping studies using Raman spectroscopy were produced by scanning areas containing at least 100 different spectra. All spectra were baseline-corrected and normalized to the most intense absorption band for comparative visualization. Reference spectral assignments were made as follows: for PCL, the characteristic ester C=O stretching (~1723 cm^{-1}), asymmetric C–O–C stretching (~1239 cm^{-1}), and CH_2 bending (~1471 cm^{-1}) bands were used as structural markers. For vancomycin, the amide I (~1650 cm^{-1}) and amide II (~1515 cm^{-1}) bands were monitored. For gentamicin, the N–H stretching (~3350 cm^{-1}) and C–N stretching (~1020 cm^{-1}) bands served as reference features. For nano-HA, the characteristic phosphate (PO_4^{3-}) stretching vibrations at ~1030 cm^{-1} and ~960 cm^{-1} were used. The potential changes in the crystalline structures of the filaments during extrusion were determined using a multipurpose X-ray Diffractometer (Bruker, D8 Advance Twin-Twin, USA).

1.2.3. Thermal properties

Following the production of composite filaments, three types of thermal analyses were conducted. Firstly, differential scanning calorimetry (DSC) analysis was performed to determine potential changes in crystal structure, melting behavior, and thermal stability resulting from the pressure and temperature experienced during filament production. Secondly, thermogravimetric analysis (TGA) was carried out in a nitrogen environment to identify degradation temperatures. Additionally, TGA experiments were performed under an air

environment, simulating the oxygen exposure that filament would face during 3D printing at maximum extrusion temperature.

TGA of samples was performed using a thermogravimetric analysis instrument (Hitachi, NEXTA STA200, Japan). Approximately 5 mg of each sample was placed in aluminum pans. The samples were then heated from 30°C to 600°C at a heating rate of 10°C/min under a flow of N_2 gas at 50 mL/min. Weight losses of the samples during heating in the N_2 environment were measured using thermogravimetric curves. Additionally, the thermal stability of the fiber samples used in extrusion steps at 200°C was determined under isothermal test conditions in ambient air. Briefly, about 5 mg samples were weighed into aluminum pans, held at 30°C for 5 min, heated to 200°C at a rate of 50°C/min, and maintained at this temperature for 5 min in an air environment (50 mL/min). After holding the samples at 200°C in the air environment, they were cooled to 30°C at a cooling rate of 50°C/min. This process simulated the conditions of extrusion and 3D printing steps while also aiming to evaluate the stability of antibiotics (vancomycin and gentamicin) used in the formulations at 200°C in an oxidative environment.

DSC analysis was performed using a Hitachi EXSTAR DSC 7020 under N_2 gas atmosphere maintained at a flow rate of 50 mL/min. Approximately 5 mg of each sample was first cooled from 25°C to -80°C at 20°C/min before undergoing a multi-stage heating process. This heating sequence included ramping from -80°C to 50°C at 15°C/min, then from 50°C to 65°C at 3°C/min, and finally reaching 170°C at a rate of 10°C/min. Following a one-minute isothermal hold at 170°C, the samples were cooled back to -80°C at 20°C/min and subjected to a second heating scan under the same conditions to determine T_g, T_m, and related enthalpies.

1.3. Determination of 3D printing conditions

The upper temperature limits were determined based on prior thermal analysis results to prevent the polymer matrix (PCL) and the integrated antibiotics (gentamicin and vancomycin) from thermal degradation. Thus, the printing conditions, including the print head temperature, build

platform temperature, and printing speed, were systematically tested to find the optimum conditions.

The porosity of 3D-printed grafts was measured using a design-based calculation from computer-aided design (CAD) geometry (nominal/theoretical porosity) using ethanol as the displacement medium.

1.4. Release behavior of drug-loaded grafts

To characterize the drug release behavior and determine the release mechanism, the experimental data were subjected to mathematical modeling using several kinetic equations. The release profiles were fitted to zero-order, first-order, Higuchi, and logarithmic empirical ($y=a \cdot \ln(x)+b$) models. The Korsmeyer–Peppas semi-empirical power law was additionally employed to determine the physical mechanism of drug transport, as shown in below.

$$M_t/M_\infty = k \cdot t^n$$

where M_t/M_∞ represents the fractional drug release at time t, k is the release rate constant, and n is the diffusion exponent. The value of n was used to determine the transport mechanism, where $n \leq 0.45$ indicates Fickian diffusion, $0.45 < n < 0.89$ indicates non-Fickian (anomalous) transport, and $n=0.89$ indicates Case II transport (zero-order release).

Model selection and the evaluation of the goodness-of-fit were performed using the coefficient of determination (r^2) and the Akaike Information Criterion (AIC). The r^2 value was used to assess the linear correlation between experimental and predicted values, while the AIC provided a measure of the relative quality of the statistical models by penalizing for the number of parameters to prevent overfitting. The model having the highest r^2 and the lowest AIC value was selected as the most appropriate to describe the drug release kinetics from the 3D-printed composite grafts. All nonlinear regression analyses and curve fittings were performed using GraphPad Prism.

2. Results

2.1. The properties of milled graft matrices

The morphology and size of PCL particles before and after the milling process are shown in Supplementary Figure 1. The initial size of PCL particles, having approximately 3 mm, was reduced to micron size after milling under cooling with liquid nitrogen, as confirmed by sieving through a 100 µm analysis sieve and Supplementary Figure 1B-C.

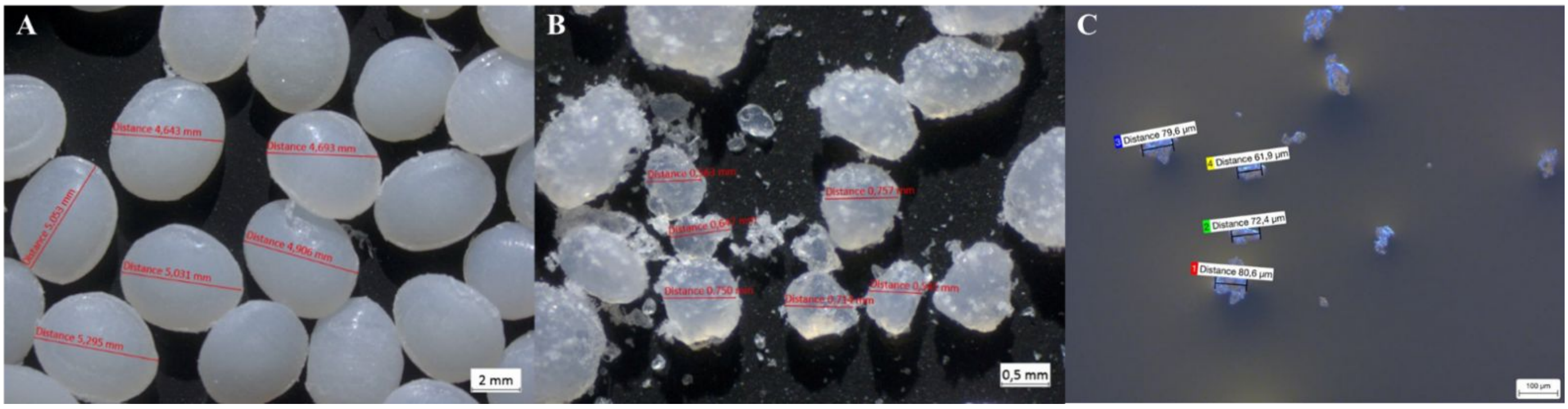

Supplementary Figure 1. Microscope images of PCL before milling (A), after pre-milling (B), and after milling (C)

The particle size measurements are shown in Supplementary Table 1, and the particle distribution of milled HA and antibiotics is presented in Supplementary Figure 2.

Supplementary Table 1. Particle size of milled samples

Sample	Size	
	Before milling	After milling
**PCL**	5 mm	0.5 mm – 70 µm (pre-milling)*
**HA**	-	197.5±14.2 nm**
**Vancomycin**	-	100.1±10.6 nm**
**Gentamicin**	-	93.1±11.6 nm**

* Obtained with sieve analysis
** Obtained with a particle size analyzer

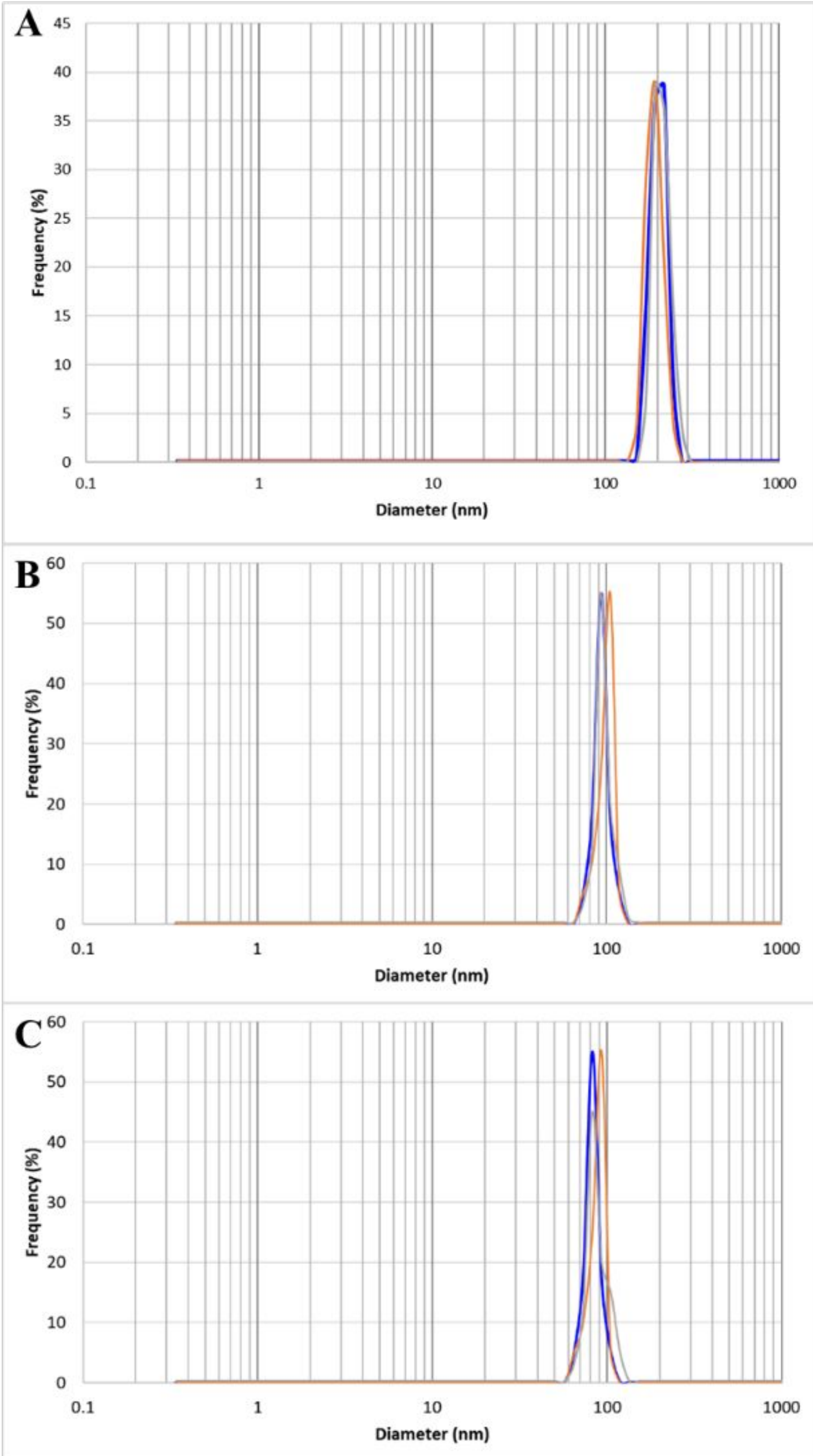

Supplementary Figure 2. Size distribution of HA (A), vancomycin (B), and gentamycin (C) after milling.

The average particle size of HA was determined as 197.5 ± 14.2 nm with a polydispersity index (PDI) of 0.423, indicating a broad particle size distribution (PDI > 0.4). The particle size and polydispersity index values for vancomycin and gentamicin were 100.1 ± 10.6 nm (PDI, 0.437) for vancomycin and 93.1 ± 11.6 nm (PDI, 0.497) for gentamicin, respectively.

FTIR spectrums of raw materials are shown in Supplementary Figure 3.

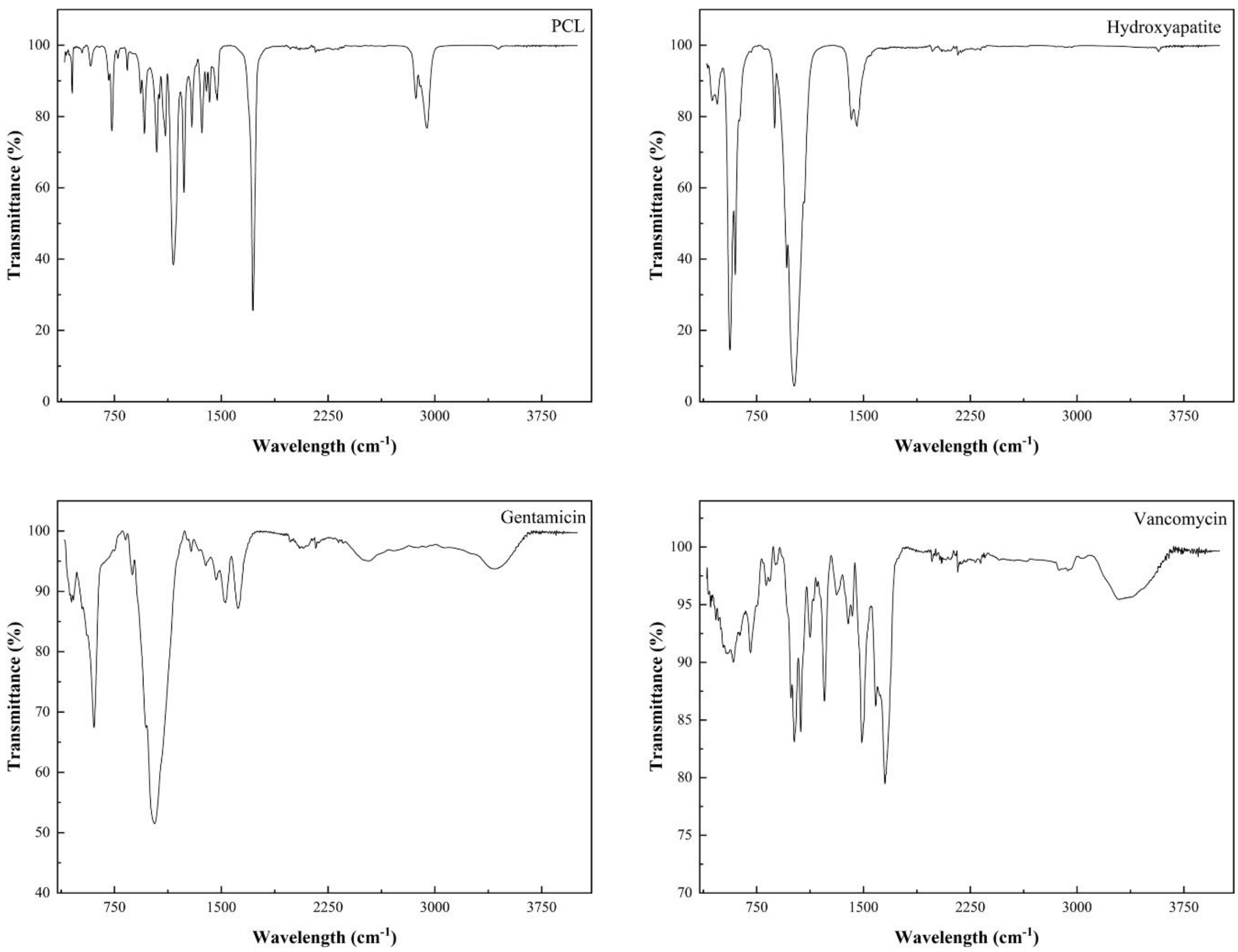

Supplementary Figure 3. FTIR spectrums of raw materials

The FTIR spectra of the raw materials (PCL, nano-HA, vancomycin, and gentamicin) confirmed their chemical identity and structural integrity. PCL displayed a strong C=O stretching band at 1720 cm^{-1} and characteristic C–H stretching peaks at 2940 cm^{-1} and 2860 cm^{-1}, while nano-HA exhibited sharp phosphate (PO_4^{3-}) P–O stretching vibrations between 1000–1100 cm^{-1} and O–P–O bending modes at 560–600 cm^{-1}. Vancomycin had distinct amide I and II bands at ~1650 cm^{-1} and ~1500–1550 cm^{-1}; and gentamicin showed broad O–H and N–H stretching bands in the 3200–3400 cm^{-1} region, confirming the preservation of all functional groups after the milling process.

XRD analyses were performed to understand the effect of grinding under nitrogen gas on the crystallite size and crystalline structure in PCL, as shown in Supplementary Figure 4.

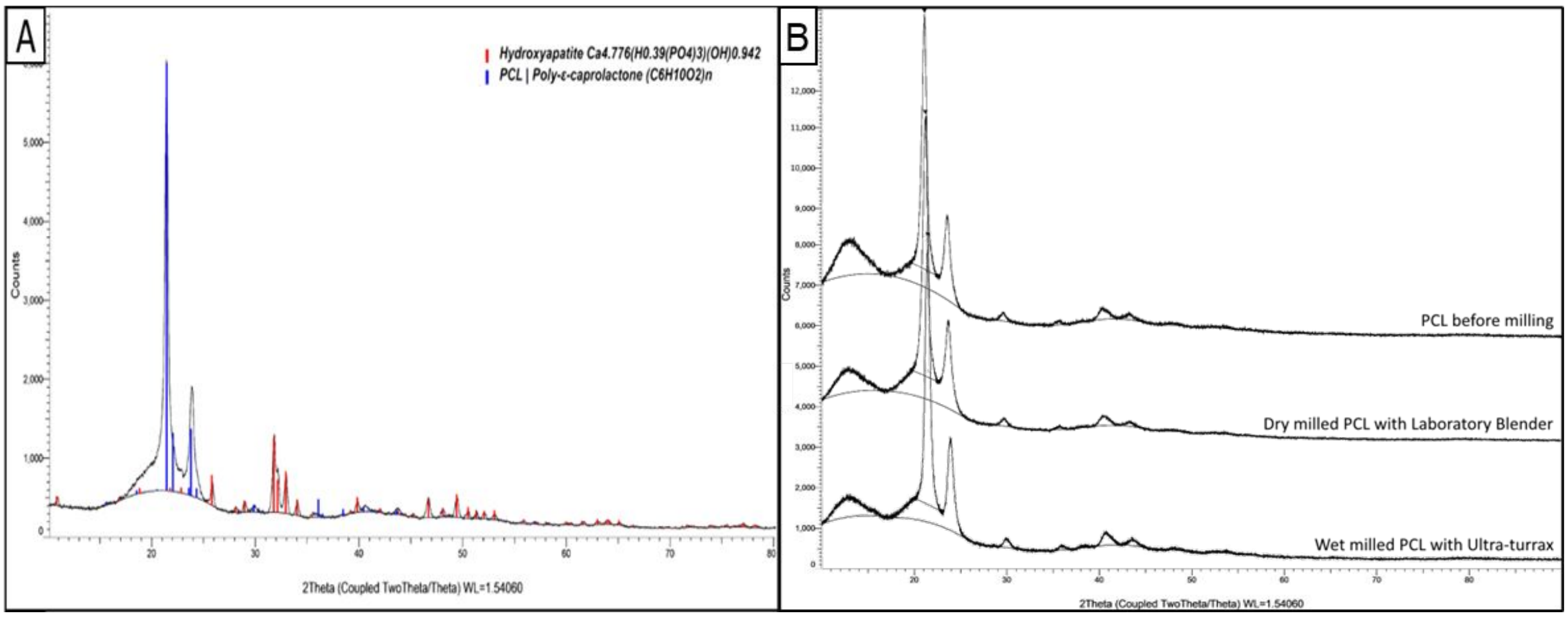

Supplementary Figure 4. XRD results of PCL and HA as a reference for the combination (A) and PCL based on the milling stages (B).

The characteristic peaks at 2θ angles were identified for PCL and HA, located at 21.4°, 22.0°, and 23.8° and identified at 25.9°, 31.8°, 32.2°, and 33.8°, respectively. The crystal size and crystalline percentage of PCL particles before and after milling are presented in Supplementary Table 2.

Supplementary Table 2. The crystallinity degree and the crystal size of PCL particles

Step	Crystal structure (%)	Crystal size (Å)
Before milling	61.5	251.5
After pre-milling	60.0	247.1
After milling	62.3	247.8

XRD results confirmed that the grinding did not have a significant effect on the crystalline and amorphous nature of PCL's structure.

2.2. The properties of drug-loaded filaments

2.2.1. Morphological and chemical properties

The representative images of the composite filaments are shown in Supplementary Figure 5.

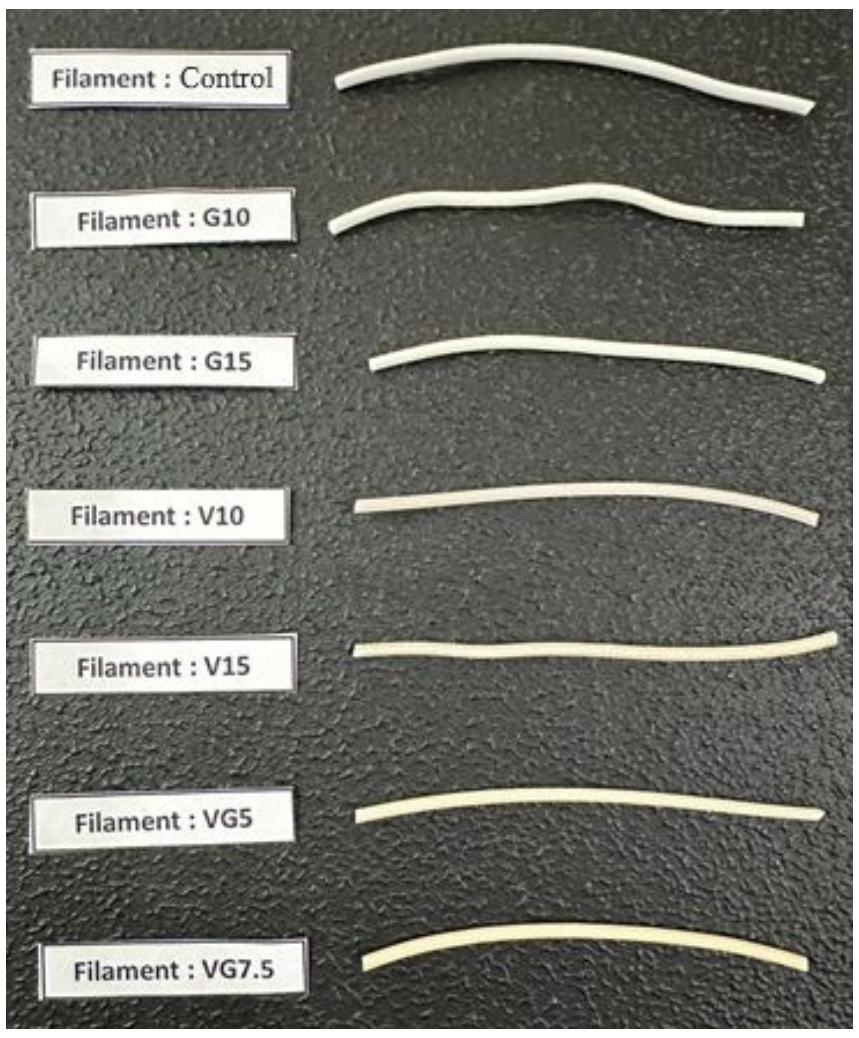

Supplementary Figure 5. The images of produced drug-loaded filaments

The AFM images indicated an increase in surface roughness with the addition of drugs to the microstructure (Supplementary Figure 6).

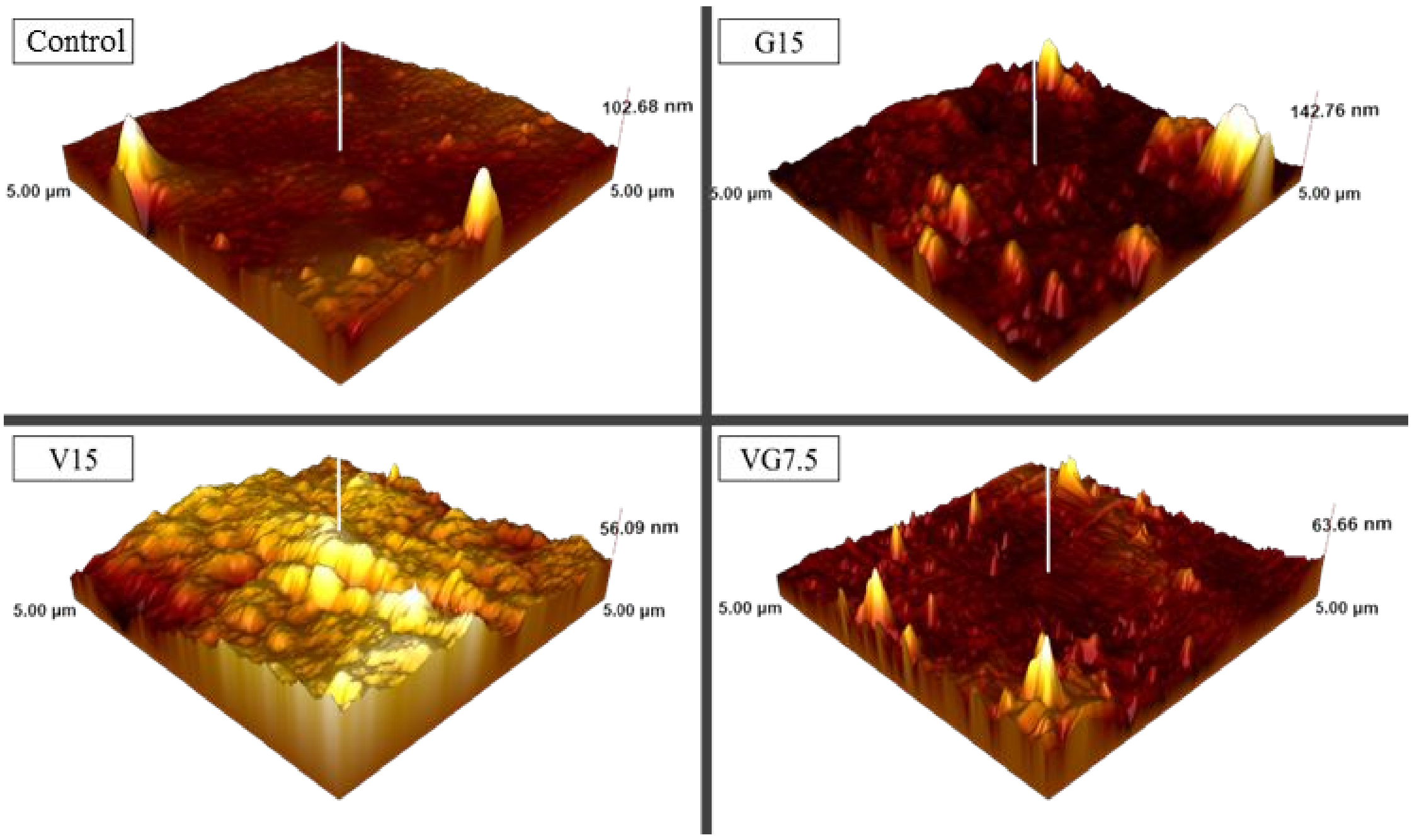

Supplementary Figure 6. AFM microscope images of composite filaments

The average surface roughness (Ra) for the control group (no drug included) was determined as 4.65 nm ± 6.92 nm, the ten-point height (Rz) was 34.08 nm, and the kurtosis (Rku) value was 18.865. For the G15 filament, the average Ra was found to be 10.52 nm ± 15.86 nm, with

an Rz of 142.21 nm and an Rku value of 12.851. The V15 filament had an average Ra of 5.01 nm ± 6.59 nm, a Rz of 54.08 nm, and a Rku value of 4.329. As for the VG7.5 filament, the average Ra was measured as 4.02 nm ± 5.31 nm, with an Rz of 61.76 nm and an Rku value of 7.121.

The effects of extrusion pressure and temperature on the incorporated drugs within the filament structure were examined through FTIR analysis. The obtained spectra indicated that the drugs did not undergo significant chemical changes during the extrusion, as shown in Supplementary Figure 7.

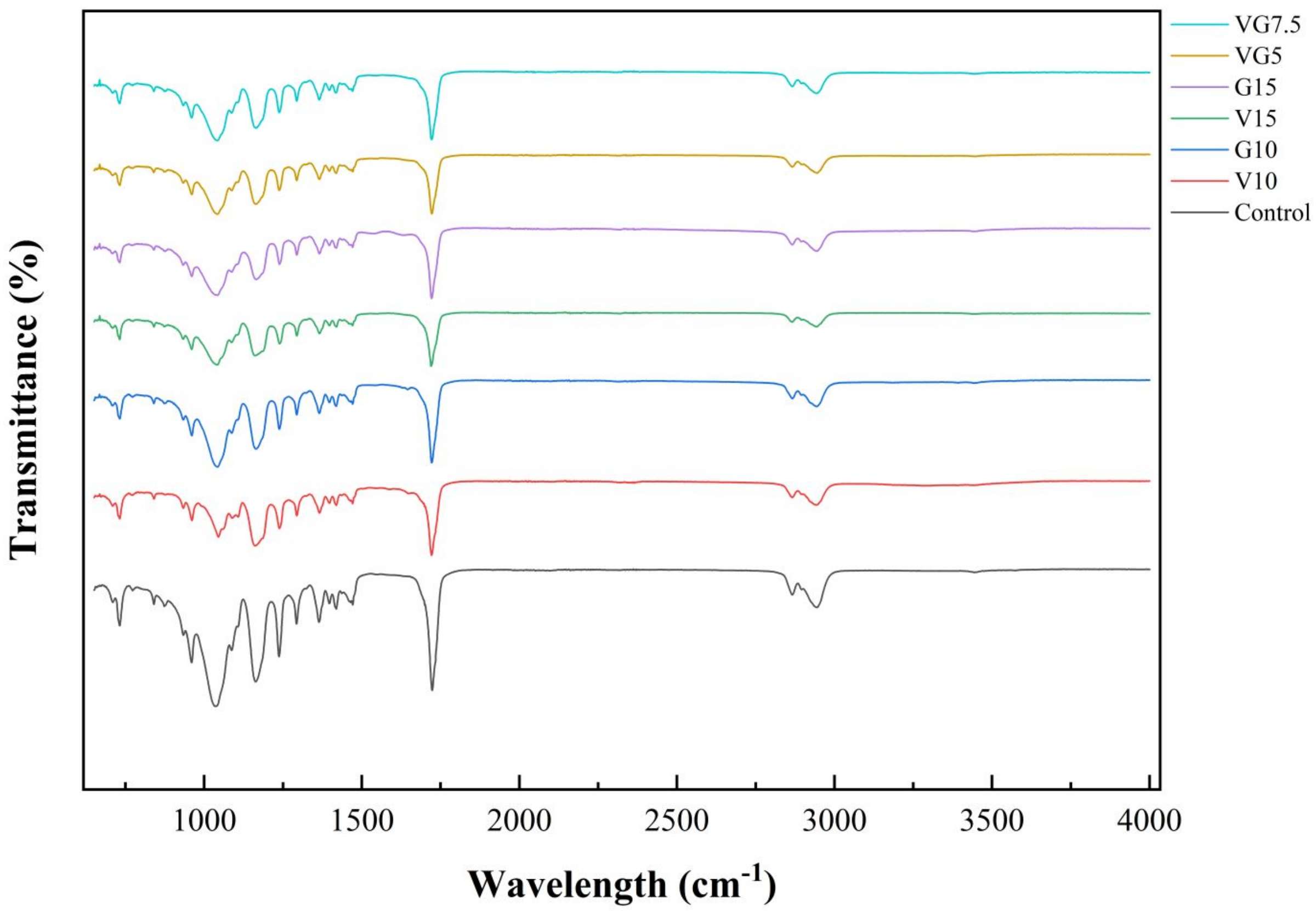

Supplementary Figure 7. FTIR spectrums of composite filaments

Peaks observed at 2850-3000 cm⁻¹, 1720-1740 cm⁻¹, 1360-1420 cm⁻¹, 1180-1260 cm⁻¹, and 1090 cm⁻¹ represent long methylene chains and alkyl groups in the polymer, the carbonyl group within the ester structure of PCL, phosphate groups within the composite, ester groups with C-

O bonds, and C-O-C bonds, respectively. Peaks attributed to the carbonyl group in the vancomycin structure might interfere with those of the carbonyl group of PCL in the 1720-1740 cm^{-1} region. Similarly, for gentamicin, interactions between amine bonds of gentamicin and the C-O (alcohol or ether) bonds of PCL might be associated with peaks observed in the 1300-1000 cm^{-1} range.

The homogeneity of drug-loaded composite filaments was assessed using the mapping technique through FTIR and Raman spectroscopy for the control group and the groups with the highest drug concentrations, namely V15, G15, and VG7.5. The 2D and 3D mapping images showed that similar spectra were obtained at every point of the composite filaments as an indication of homogeneous composites (Supplementary Figure 8). The variations in spectrum intensity between FTIR and Raman maps were observed in the green, red, and blue regions, which might be attributed to changes in surface roughness based on both spectrum scans and component analysis. Together, these FTIR spectral observations confirm that the functional groups of both vancomycin and gentamicin are preserved within the composite filament matrix following melt extrusion at 170–180°C and that no new covalent bonds or irreversible chemical interactions between the drug molecules and the PCL/HA matrix were detected under the processing conditions applied.

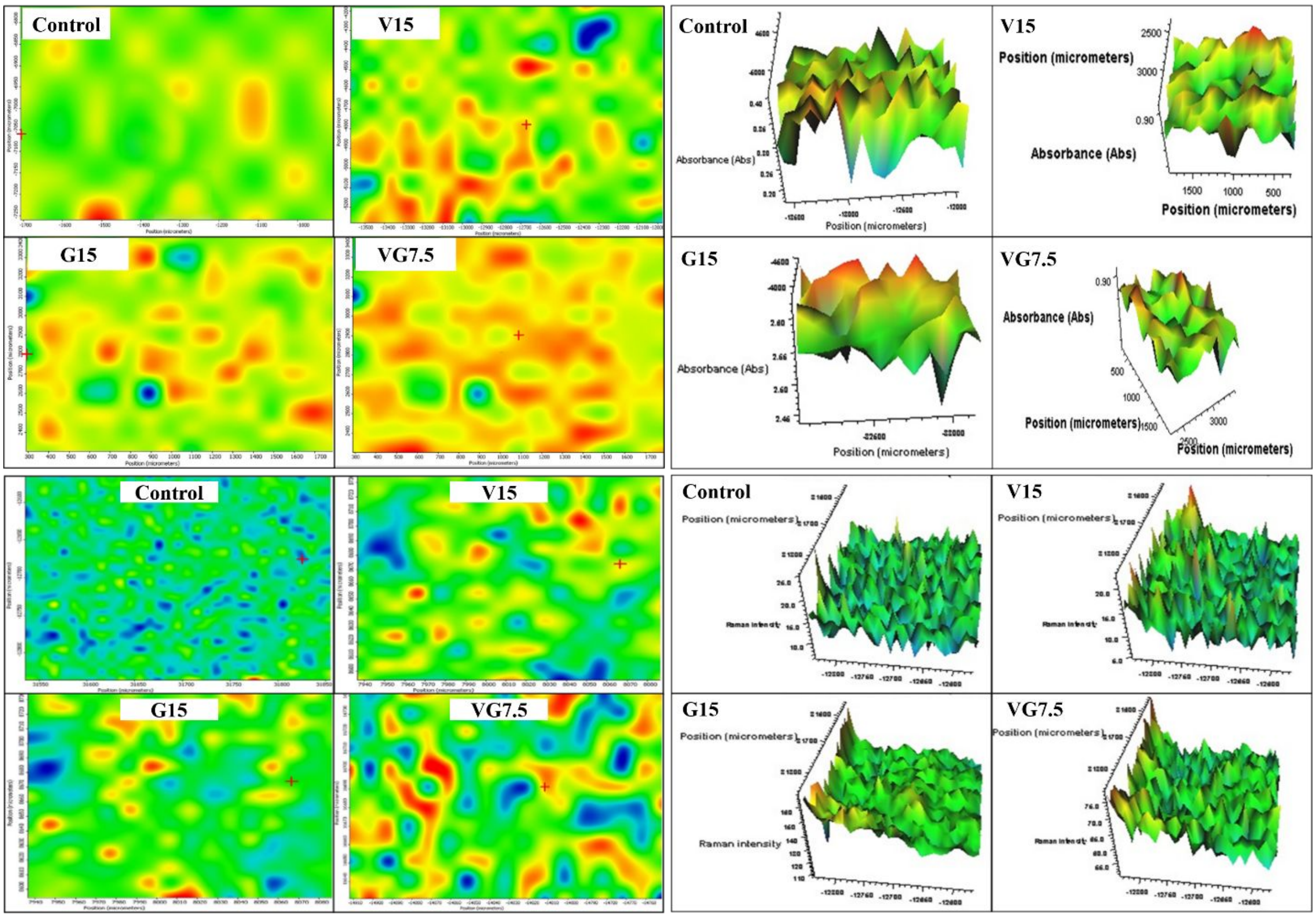

Supplementary Figure 8. FTIR (A and B) and Raman (C and D) 2D mapping (left) and 3D mapping (right) spectroscopy with a resolution of 1.7 µm.

XRD analyses were performed to examine potential changes in the crystal structures of the composite filaments due to the temperature and pressure exposure. XRD patterns indicated that the extrusion step did not induce any noticeable alterations in the crystal and amorphous structures of PCL (Supplementary Figure 9). Typical peaks for PCL were identified at 21.4°, 22.0°, and 23.8°, while characteristic peaks for HA were observed at 25.9°, 31.8°, 32.2°, and 33.8°.

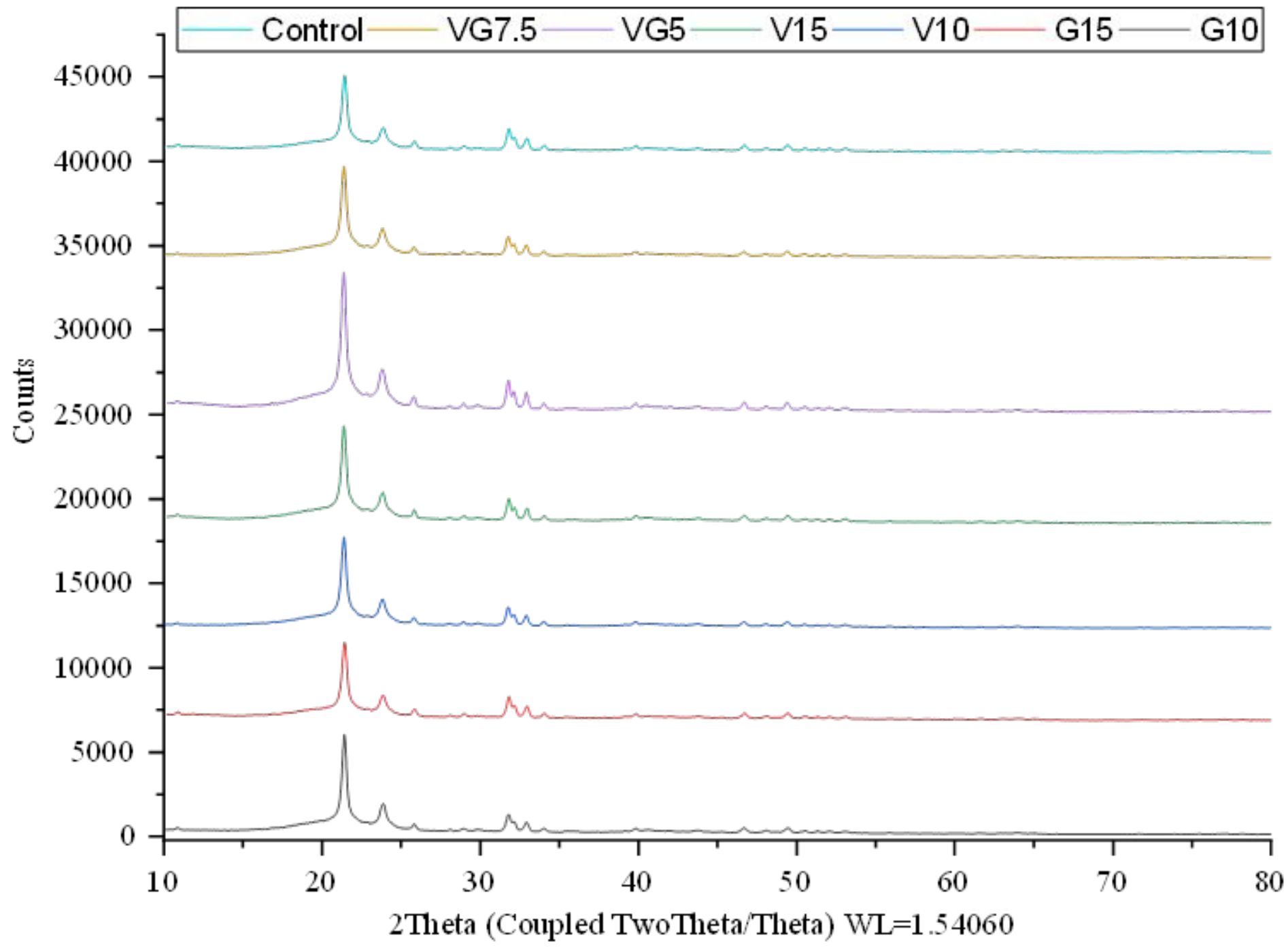

Supplementary Figure 9. XRD patterns of composite filaments

The degree of crystallinity and the crystal size observed for composite filaments are shown in Supplementary Table 3.

Supplementary Table 3. Crystallinity and crystal size of filaments

Sample	Crystallinity (%)	Crystal size (Å)
Control	61.5	246.3
V10	60.0	243.1
V15	62.3	243.2
G10	59.8	248.2
G15	60.1	247.1
VG5	61.3	244.4
VG7.5	60.5	245.3

2.2.2. Thermal properties of filaments

The thermal stability of composite filaments, including initial degradation, degradation temperature, and residual weight, is given in Supplementary Table 4.

Supplementary Table 4. Thermal characteristics of composite filaments

Sample	T_{onset} (°C)	Degradation temperature (°C)

		1st degradation	2nd degradation	Residual weight (%)
**Control**	324.39±0.54[e]	-	364.09±2.00[b]	29.38±0.04[a]
**V10**	387.48±0.67[b]	-	412.75±0.11[a]	26.81±0.09[d]
**V15**	374.48±0.68[d]	209.27±0.37[b]	403.75±0.35[a]	28.83±0.09[b]
**G10**	385.30±0.42[c]	254.17±0.23[a]	411.09±0.12[a]	11.78±0.08[g]
**G15**	393.04±0.05[a]	257.25±0.35[a]	415.34±0.47[a]	13.84±0.08[f]
**VG5**	384.17±0.24[c]	255.50±0.71[a]	409.42±0.59[a]	25.95±0.03[e]
**VG7.5**	384.31±0.43[c]	255.25±0.35[a]	409.17±0.23[a]	27.98±0.14[c]

Results indicated with different letters in the same column are statistically different according to Tukey's multiple comparison test ($p < 0.05$).

The lowest degradation temperature was observed for V15 (209.27°C). While T_{onset} of G10, G15, VG5, and VG7.5 filaments were approximately 255°C, V10 had a single degradation temperature of 412.75°C. The absence of an initial degradation temperature around 209°C in V10 filament, in contrast to V15, might be due to the encapsulating ability of PCL for vancomycin at relatively low amounts. TGA and derivative thermogravimetric (DTG) curves are shown in Supplementary Figure 10.

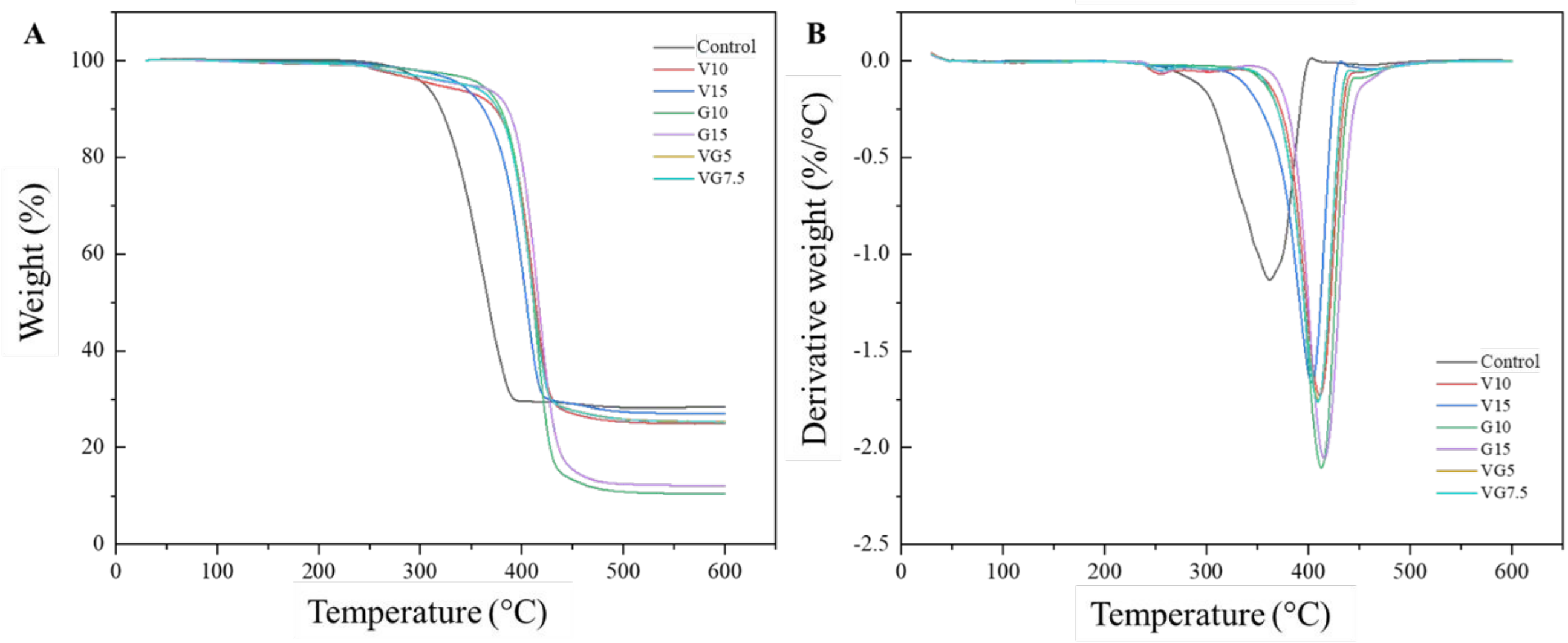

Supplementary Figure 10. TGA (A) and DTG (B) curves of composite filaments

Despite previous TGA analyses determining the thermal stability and degradation temperatures of extruded drug-loaded composite filaments, these analyses conducted in a nitrogen environment are not suitable for evaluating potential degradation profiles in the air environment

encountered during 3D printing. Hence, analyses were also conducted using the air environment mimicking the conditions a filament would experience during printing at the maximum extrusion temperature. The results indicated that both the filaments and the encapsulated drugs retained their thermal stability throughout and after 3D printing in the air environment at maximum extrusion temperature. TGA curves obtained for those samples which exposed to air are provided in Supplementary Figure 11.

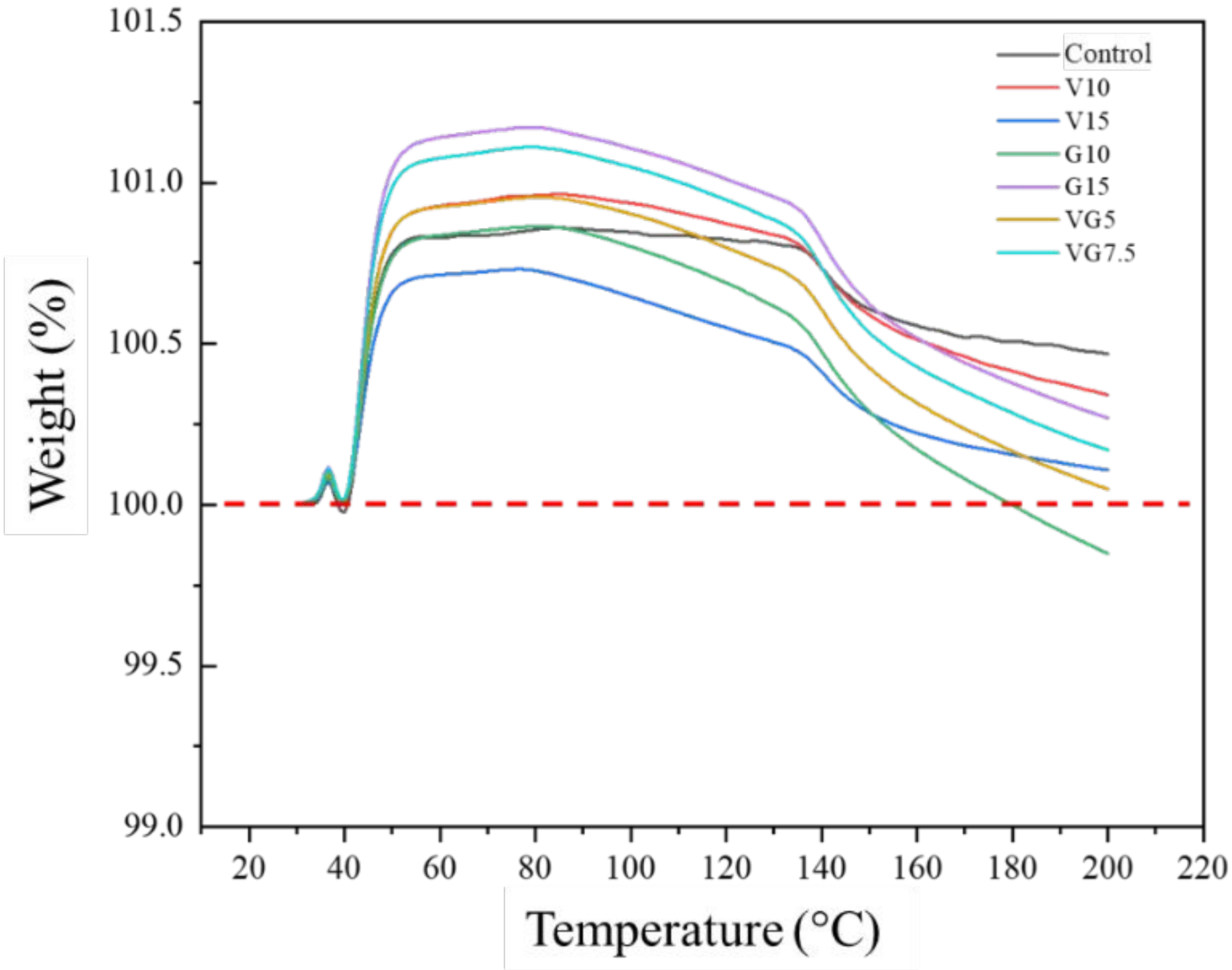

Supplementary Figure 11. The weight changes in samples kept in air conditions at 200°C

Thermal properties, including T_g (°C), ΔH_m (J/g), and T_m (°C) are presented in Supplementary Table 5.

Supplementary Table 5. Thermal properties of composite filaments

Sample	T_g (°C)	ΔH_m (J/g)	T_m (°C)
**Control**	-61.97±0.71[a]	22.20±0.59[b]	57.81±0.24[a]
**V10**	-59.53±0.34[a]	26.99±0.65[a]	57.31±0.21[a]
**V15**	-60.71±1.76[a]	22.65±0.06[b]	57.56±0.18[a]
**G10**	-60.97±1.77[a]	22.48±0.07[b]	57.94±0.18[a]
**G15**	-60.96±1.44[a]	27.58±0.04[a]	57.33±0.20[a]
**VG5**	-59.36±0.23[a]	22.37±0.26[b]	57.95±0.21[a]
**VG7.5**	-60.61±1.56[a]	21.69±0.16[b]	57.29±0.21[a]

Results indicated with different letters in the same column are statistically different according to Tukey's multiple comparison test ($p < 0.05$).

The thermal characteristics of PCL were similar to those composite filaments, indicating that extrusion did not affect the thermal profile. DSC curves of the second cycle analysis are presented in Supplementary Figure 12.

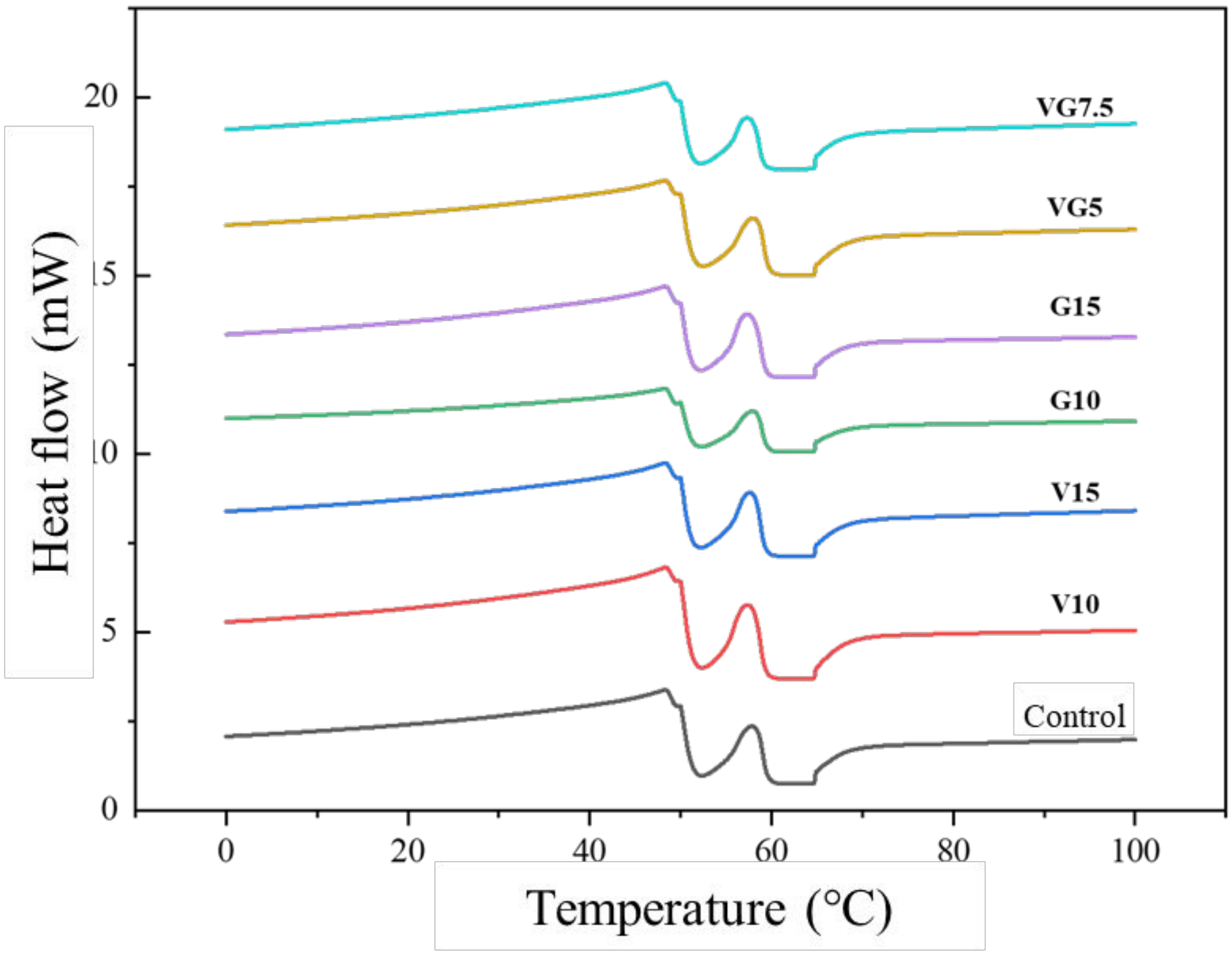

Supplementary Figure 12. DSC curves (2nd heating cycle) of composite filaments

2.3. Optimum conditions for the 3D printing process and characterization of grafts

The preliminary study results of the 3D printing process and related explanations are shown in Supplementary Table 6.

Supplementary Table 6. 3D printing parameters

Print temperature (°C)	Print rate (mm/s)	Table temperature (°C)	Explanation
**135**	30	25	Print Failed, No Filament Flow: Printing could not be completed due to a lack of filament flow.
**150**	30	25	Print Failed, Irregular Filament Flow: Printing was unsuccessful due to inconsistent filament flow.
**170**	30	25	Print Failed, Filament Did Not Adhere to Bed: Printing was unsuccessful, as the filament did not adhere to the print bed.
**170**	30	45	Print Failed, Warping Due to Insufficient Cooling: Printing could not be completed as the layers did not cool adequately, resulting in distortion of the model.
**170**	15	45	Models Printed Successfully: The models were printed without any issues.
**180**	30	45	Print Failed, Warping Due to Insufficient Cooling: Printing was unsuccessful as the layers did not cool sufficiently, leading to model distortion.
**180**	15	45	Models Printed Successfully: The models were successfully printed.
**190**	30	45	Print Failed, Slight Color Variation: Printing was unsuccessful, with a slight color variation observed.

Temperatures below 150°C resulted in irregular or absent filament flow, while printing at speeds of 30 mm/s led to structural deformations due to insufficient layer cooling. The optimal printing parameters were determined as the lowest temperature point that ensured consistent extrudability and structural stability. 170°C, 45°C, and 15 mm/s were chosen as optimal points for print head (nozzle) temperature, build platform (bed) temperature, and print rate,

respectively. These parameters were applied for uniform production of control and drug-loaded composite grafts.

2.4. Determination of release mechanism of drugs from grafts

The details on mass spectrometric quantification are presented in Supplementary Table 7.

Supplementary Table 7. LC-MS/MS method validation parameters for gentamicin and vancomycin quantification

Parameter	Gentamicin	Vancomycin
MRM Transition (precursor → product, m/z)	450.3 → 160.1/322.2 (C1a)<br>464.3 → 160.1/322.2 (C2)<br>478.2 → 157.1/322.2 (C1)	1450.8 → 1145.5<br>1450.8 → 1307.8
Linearity range (µg/mL)	10, 20, 50, 100	10, 20, 50, 100
R^2 (calibration curve)	> 0.999	> 0.999
Intra-day precision (RSD%, n=6)	2.6%	3.5%
Inter-day precision (RSD%, n=18)	5.3%	7.8%
Accuracy (% recovery, n=6)	97.8%	101.1%

The calibration curves for vancomycin, gentamicin C1, gentamicin C1a, and gentamicin C2 are presented in Supplementary Figure 13.

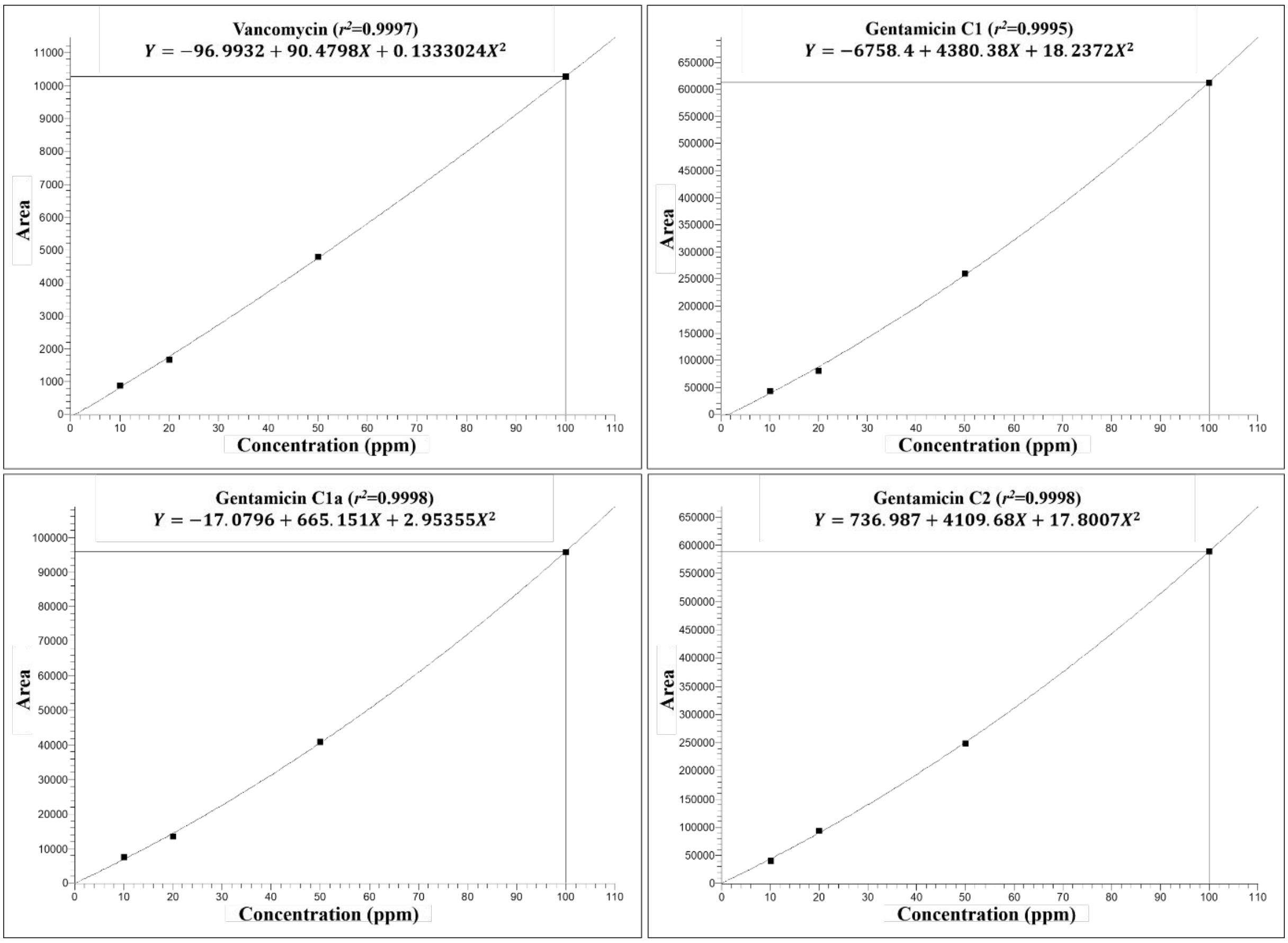

Supplementary Figure 13. Calibration curves of vancomycin and gentamicin isoforms constructed using 2nd-degree polynomial regression

Cumulative release data (21-day) were fitted to four candidate models, and the coefficient of determination (r^2) and AIC values are shown in Supplementary Table 8. The model with the highest r^2 and lowest AIC was selected as the best fit.

Supplementary Table 8. Kinetic model fitting parameters for drug release from grafts

Sample	Cumulative release rate (%)	Zero-Order r^2	First-Order r^2	Higuchi r^2	Logarithmic r^2	AIC (best fit)	KP* *n*
V10	30.02	0.4535	0.9666	0.9640	0.9899	3.03	0.414
V15	26.01	0.4320	0.9664	0.9601	0.9893	1.65	0.410
G10	66.18	0.3668	0.9582	0.9493	0.9886	12.90	0.424
G15	61.87	0.4055	0.9621	0.9556	0.9884	12.39	0.435
VG5	48.84	0.3825	0.9612	0.9523	0.9903	8.41	0.399
VG7.5	40.34	0.3553	0.9689	0.9426	0.9909	5.86	0.395

r2 values from curve fitting; KP = Korsmeyer-Peppas; n = release exponent. * KP mechanism as Fickian diffusion
